# *In vitro* reconstitution of a minimal human centrosome scaffold capable of forming and clustering microtubule asters

**DOI:** 10.1242/jcs.264121

**Published:** 2025-06-27

**Authors:** Manolo U. Rios, Weronika E. Stachera, Nicole E. Familiari, Claudia Brito, Thomas Surrey, Jeffrey B. Woodruff

**Affiliations:** ^1^ Department of Cell Biology, UT Southwestern Medical Center, Dallas, TX 75390, USA; ^2^ Centre for Genomic Regulation (CRG), Barcelona Institute of Science and Technology (BIST), 08003 Barcelona, Spain; ^3^ Universitat Pompeu Fabra (UPF), 08002 Barcelona, Spain; ^4^ Catalan Institution for Research and Advanced Studies (ICREA), 08010 Barcelona, Spain

**Keywords:** PCM, Centrosome, CDK5RAP2, Microtubule, Self-assembly, Scaffold

## Abstract

CDK5RAP2 (also known as CEP215) is a key pericentriolar material (PCM) protein that recruits microtubule-nucleating factors at human centrosomes. Here, using an *in vitro* reconstitution system, we show that CDK5RAP2 is sufficient to form micron-scale scaffolds using nanometer-scale nucleators in a PLK-1-regulated manner. CDK5RAP2 assemblies recruited and activated γ-tubulin ring complexes (γ-TuRCs) which, in the presence of α/β-tubulin, generated microtubule asters. We found that amino acid F75 in CDK5RAP2 helps to recruit γ-TuRC and is indispensable for γ-TuRC activation. Furthermore, our system recapitulated key features of centrosome-amplified cancer cells. CDK5RAP2 scaffolds recruited the molecular motor HSET (also known as KifC1), which enhanced concentration of α/β-tubulin, microtubule polymerization and clustering of the assemblies. Our results highlight the specificity and selectivity of *in vitro*-generated CDK5RAP2 scaffolds, and identify a minimal set of components required for human PCM assembly and function. This minimal model offers a powerful tool for studying centrosome biology and dysfunction in human health and disease.

## Teaser

**Summary:** Purified human proteins are used to define minimal modules for centrosome scaffold assembly and microtubule aster nucleation.

## INTRODUCTION

Centrosomes are major microtubule-organizing centers (MTOCs) in animal cells. These membraneless organelles play a crucial role in mitotic cell division and development (Conduit et al., 2015; Pimenta-Marques and Bettencourt-Dias, 2020). Centrosomes form through structured centrioles that organize a micron-scale layer of protein called pericentriolar material (PCM) that nucleates and anchors microtubules. One of the main proteins underlying PCM formation is CDK5 regulatory subunit-associated protein 2 (CDK5RAP2), also known as CEP215 (Ching et al., 2000; Fong et al., 2008). Dysfunction of CDK5RAP2 can lead to genomic instability, infertility, microcephaly and other developmental disorders (He et al., 2024; Megraw et al., 2011; Nasser et al., 2020; Rahimian et al., 2023; Yigit et al., 2015).

Structural studies of CDK5RAP2 and its functional homologs in flies and worms (Cnn and SPD-5, respectively) show that centrosome assembly is typically achieved in two general steps. First, these proteins are nucleated around the centrioles to form the interphase PCM (Cabral et al., 2019; Fu and Glover, 2012; Hamill et al., 2002; Lawo et al., 2012; Mennella et al., 2012). In humans, nucleation of CDK5RAP2 around the centrioles is mediated by the adaptor protein pericentrin (Buchman et al., 2010; Wang et al., 2010). Pericentrin decorates the outer wall of the centrioles and binds the conserved CM2 domain in CDK5RAP2 located at its C-terminus [amino acids (a.a.) 1680–1893] (Buchman et al., 2010; Kim and Rhee, 2014; Lawo et al., 2012). However, recent evidence shows that CDK5RAP2 can assemble into micron-scale scaffolds in the absence of centrioles (Chen et al., 2022; Watanabe et al., 2020). This suggests that centrioles are not strictly required to nucleate CDK5RAP2 assembly. The universal requirements for PCM nucleation thus require further investigation.

The second major step in centrosome assembly involves increased accumulation of CDK5RAP2 or its functional homologs in preparation for mitosis, also termed centrosome expansion. This process is regulated by mitotic kinases, such as polo-like kinase 1 (PLK-1); in flies and worms, PLK-1 phosphorylation sites on major PCM proteins are known (Conduit et al., 2014; Decker et al., 2011; Dobbelaere et al., 2008; Feng et al., 2017; Sunkel and Glover, 1988; Woodruff et al., 2015). In *D. melanogaster*, centrioles help generate local pulses of PLK-1 activity to initiate PCM expansion (Feng et al., 2017; Wong et al., 2022). Our group and others have demonstrated that PLK-1 phosphorylation induces conformational changes in SPD-5 that promotes its multimerization in the presence or absence of centrioles (Mittasch et al., 2020; Ohta et al., 2021; Rios et al., 2024; Woodruff et al., 2015; Wueseke et al., 2016). PLK-1 is also necessary for centrosome expansion in human cells (Haren et al., 2009; Santamaria et al., 2011). However, a comprehensive list of PLK-1 phosphorylation sites in CDK5RAP2 and identification of which drive centrosome expansion does not currently exist. Also, it is not known whether CDK5RAP2 can multimerize in a PLK-1-dependent fashion without centrioles.

CDK5RAP2 has been proposed to serve as a major PCM scaffold due to its ability to form a stable, salt-resistant network within centrosomes and recruit numerous centrosome-associated proteins (Andersen et al., 2003; Buchman et al., 2010; Ching et al., 2000; Fong et al., 2008, 2009; Graser et al., 2007). CDK5RAP2 plays a pivotal role in recruiting γ-tubulin ring complexes (γ-TuRCs) to the centrosomes (Choi et al., 2010; Fong et al., 2008). γ-TuRCs promote microtubule nucleation by forming a template for the start of microtubule growth (Brito et al., 2024), thus contributing to the main function of a centrosome as an MTOC. γ-TuRC anchoring is thought to be mediated by the conserved CM1 domain of CDK5RAP2 located at its N-terminus (a.a. 51–100) (Choi et al., 2010). Phenylalanine 75 (F75) in the CM1 domain of CDK5RAP2 is particularly important for its association with γ-TuRCs (Choi et al., 2010). Furthermore, *in vitro* experiments have shown that CDK5RAP2 strongly stimulates the nucleation of microtubules from γ-TuRCs via its CM1 domain (Rai et al., 2024; Serna et al., 2024; Xu et al., 2024). However, these experiments did not recapitulate microtubule aster formation, raising the possibility that CDK5RAP2 self-assembly into a micron-scale scaffold is required for that function.

In addition to γ-TuRCs, CDK5RAP2 interacts with the minus-end-directed motor HSET (also known as KifC1), a member of the kinesin-14 family (Chavali et al., 2016). In a wild-type context, HSET is required to keep centrosomes attached to the mitotic spindle poles. In centrosome-amplified cancer cells, HSET is necessary to cluster supernumerary centrosomes into pseudo-bipolar spindles and promote cancer cell survival (Chavali et al., 2016). Other studies have shown that HSET is sufficient to cluster microtubules and that IFT proteins, in complex with HSET, promote clustering (Hentrich and Surrey, 2010; Norris et al., 2018; Roostalu et al., 2018; Vitre et al., 2020). However, the minimal components sufficient to achieve centrosome clustering remain unclear.

To deepen our understanding of CDK5RAP2 nucleation, supramolecular scaffold assembly and regulation, and microtubule organizing function, we developed a novel *in vitro* reconstitution system using purified human proteins. We found that purified CDK5RAP2 self-assembles into micron-scale scaffolds in the presence of crowding agents or when locally concentrated by a pentameric antibody (IgM) targeting the CM2 domain. This system offers a robust assay to explore the regulatory interactions of CDK5RAP2, as the CDK5RAP2 assemblies can be modulated by human PLK-1, which enhances assembly and nucleation. Using proteomics, we mapped PLK-1 phosphorylation sites in full-length CDK5RAP2. Additionally, the reconstituted CDK5RAP2 assemblies were capable of nucleating microtubule asters in the presence of purified α/β-tubulin and human γ-TuRCs. The specificity and selectivity of this feature was validated using a CDK5RAP2(F75A) mutant, revealing that CDK5RAP2 macromolecular scaffolds by themselves cannot bind α/β tubulin. *In vitro*-generated CDK5RAP2 scaffolds also recruited HSET, but not a mutant lacking its N-terminal intrinsically disordered region (IDR). Our results support the functional relevance of HSET in clustering centrosomes and organizing microtubule arrays *in vitro*.

## RESULTS

### Purified human CDK5RAP2 can assemble into micron-scale scaffolds *in vitro*

**Fig. 1.**
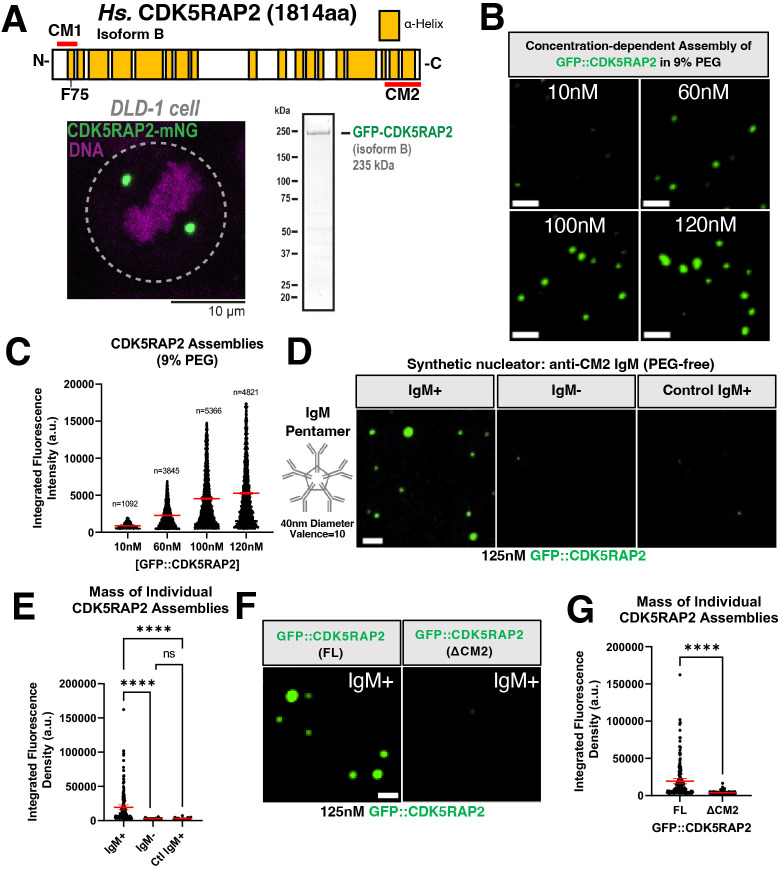
**Purified human CDK5RAP2 is sufficient to form micron-scale scaffolds.** (A) Linear representation of *Homo sapiens* (*Hs.*) CDK5RAP2 (CEP215) isoform B, human colon cancer DLD-1 cell expressing CDK5RAP2–mNeonGreen (mNG) and purified GFP::CDK5RAP2 isoform B from SF9 insect cells (images representative of three repeats). Dashed lines highlight cell edge. Yellow blocks in linear CDK5RAP2 diagram represent α-helical regions predicted by the AlphaFold Protein Structure Database. CM1 (a.a. 51–100) and CM2 (a.a. 1715–1814) domains are indicated by red bars. The CM1 domain contains F75. (B) Purified GFP::CDK5RAP2 combined with 9% PEG at various concentrations (10 nM, 60 nM, 100 nM and 120 nM). Scale bars: 5 μm. (C) Quantification of results as in B [mean±95% c.i.; 10 nM (*n*=1092 assemblies), 60 nM (*n*=3845 assemblies), 100 nM (*n*=5366 assemblies), 120 nM (*n*=4821 assemblies]. (D) GFP::CDK5RAP2 assemblies were nucleated by adding <11 pM IgM raised against the CM2 domain of CDK5RAP2 (IgM+). Addition of buffer with no IgM (IgM−) or an anti-myosin heavy chain (control IgM) did not nucleate micron-scale GFP::CDK5RAP2 assemblies. Scale bar: 5 μm. (E) Quantification of results as in D [mean±95% c.i.; IgM+ (*n*=166 assemblies), IgM− (*n*=23 assemblies), control IgM (*n*=20 assemblies)]. *P*-values from Kruskal–Wallis test followed by Dunn's multiple comparisons test. (F) Assembly of full length (FL) GFP::CDK5RAP2 into micron-scale scaffolds (left panel). GFP::CDK5RAP2 lacking the CM2 domain (ΔCM2) does not form micron-scale assemblies (right panel). This domain (last 200 a.a.) is recognized by the IgM. Scale bar: 5 μm. (G) Quantification of results as in F. Data points represent individual GFP::CDK5RAP2 assemblies (mean±95% c.i.; FL, *n*=166 assemblies; ΔCM2, *n*=131 assemblies). *P*-values from Mann–Whitney test. Error bars are sometimes too small to be visible. ****P<0.0001; ns, not significant. a.u., arbitrary units.

In mitotic human cells, CDK5RAP2 molecules accumulate around centrioles to build a spherical structure 1–2 µm in diameter (Fig. 1A) (Fong et al., 2008; Lawo et al., 2012). CDK5RAP2 is expressed as multiple isoforms. AlphaFold2 predicts that the longest isoform encodes a 215-kDa protein comprising 19 α-helical regions interspersed by intrinsically disordered linkers (Jumper et al., 2021; Varadi et al., 2021, 2023). CDK5RAP2 isoform B (Protein ID Q96SN8-4, lacking a.a. 1576–1654) is a dominant splice variant expressed in HeLa and colon cancer cells (Harrison et al., 2018; He et al., 2024; Hein et al., 2015). To investigate whether CDK5RAP2 can self-assemble into a multimeric scaffold, we first expressed and purified GFP-tagged CDK5RAP2 isoform B from SF9 cells (Fig. 1A). At near-physiological salt and protein concentrations (10–120 nM protein, 150 mM KCl; Hein et al., 2015) GFP::CDK5RAP2 was soluble and did not form micron-scale scaffolds (Fig. S1A). We hypothesized that to multimerize *in vitro*, CDK5RAP2 might require conditions that more closely resemble the intracellular environment. First, we mimicked the crowded environment inside living cells using molecular crowding agents. Using as little as 3% (w/v) polyethylene glycol 3000 (PEG), GFP::CDK5RAP2 formed micron-scale assemblies *in vitro* (Fig. S1A,B). The size and number of these assemblies scaled positively with the concentration of CDK5RAP2 (Fig. 1B,C) and PEG (Fig. S1B). Other crowding agents [lysozyme, polyvinylpyrrolidone (PVP), dextran and Ficoll) also induced CDK5RAP2 self-assembly (Fig. S1C,D). Thus, macromolecular crowding, and not PEG per se, is sufficient to promote CDK5RAP2 assembly into micron-scale structures.

In cells, CDK5RAP2 assembles around the centrioles and not everywhere in the cytoplasm. Although effective, use of synthetic crowders to induce CDK5RAP2 multimerization does not recapitulate this regulated assembly. Thus, we devised an alternative way to generate micron-scale CDK5RAP2 assemblies*.* CDK5RAP2 requires its CM2 domain to localize around centrioles (Wang et al., 2010). Thus, we tested the effect of introducing a pentameric IgM antibody (valence=10 binding sites) raised against the CM2 domain of CDK5RAP2. We hypothesized that this antibody could locally concentrate CDK5RAP2 and lower the energy barrier for CDK5RAP2 assembly, thus mimicking centriole-based nucleation *in vivo*. Indeed, in the presence of <11 pM anti-CM2 CDK5RAP2 IgM, we observed spherical, micron-scale GFP::CDK5RAP2 assemblies (Fig. 1D,E). No assemblies formed when IgM was absent or when a control anti-myosin heavy chain IgM was used (Fig. 1D,E). For the antibody to produce robust assemblies, it was essential to reduce the concentration of KCl to 50 mM. Increasing KCl to 275 mM led to the disassembly of pre-formed assemblies, indicating reversibility (Fig. S1E). However, CDK5RAP2 assemblies remained stable after rapid dilution into a 50 mM KCl buffer (Fig. S1E) and displayed no internal or external turnover after photobleaching, similar to what was observed in cultured cells treated with nocodazole (Jia et al., 2013) (Fig. S1F). Thus, this simple *in vitro* module recapitulates three key features of *in vivo* PCM-localized CDK5RAP2 – controlled nucleation, reversibility and low dynamics.

To further test the specificity and assembly capabilities of the CDK5RAP2 antibody, we compared the assembly of GFP::CDK5RAP2 lacking its CM2 domain (ΔCM2) with full-length protein. Only full-length GFP::CDK5RAP2 formed robust micron-scale assemblies in the presence of the IgM nucleator (Fig. 1F,G). Overall, our observations demonstrate the ability of CDK5RAP2 to multimerize in the presence of crowding agents or a synthetic nucleator. Furthermore, our results reinforce *in vivo* findings demonstrating that centrioles are not strictly required for CDK5RAP2 micron-scale assembly (Chen et al., 2022).

### PLK-1 phosphorylation increases the size of CDK5RAP2 scaffolds *in vitro*

**Fig. 2.**
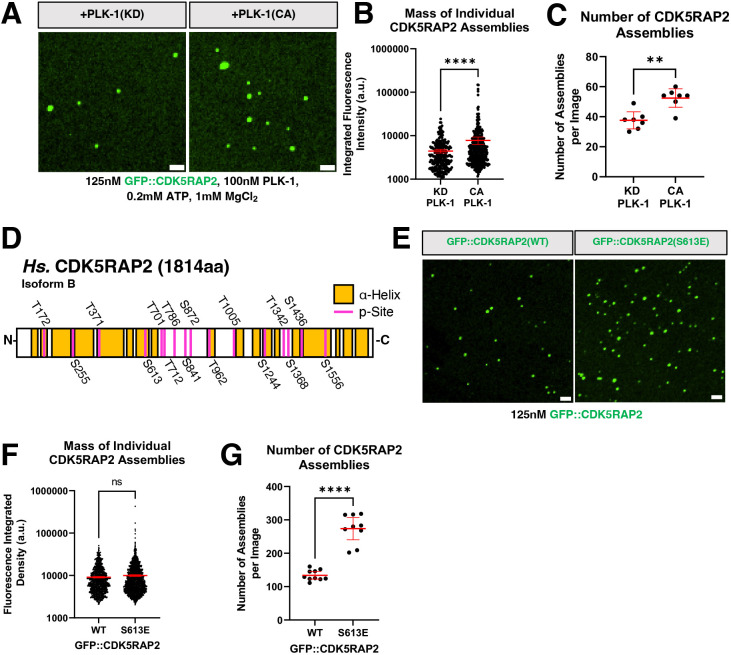
**Phospho-regulation of *in vitro* CDK5RAP2 scaffolds.** (A) GFP::CDK5RAP2 assemblies generated in the presence of purified kinase dead (KD) or constitutively active (CA) human PLK-1. Scale bars: 5 μm. (B) Quantification of results as in A. Data points represent masses of individual GFP::CDK5RAP2 assemblies [mean±95% c.i.; KD PLK-1 condition (*n*=604 assemblies), CA PLK-1 condition (*n*=511 assemblies)]. Significant differences were assessed using a Mann–Whitney test. (C) Total number of assemblies per image from experiments as in A. Each data point represents the total number of assemblies per image (mean±95% c.i.; KD *n*=7, CA *n*=7). Significant differences were assessed using a Mann–Whitney test. (D) Identified PLK-1 phosphorylation sites (p-Sites) in GFP::CDK5RAP2 incubated with human PLK-1 plus ATP·MgCl2. Control reactions consisted of pre-dephosphorylated GFP::CDK5RAP2 plus PLK-1 but no ATP·MgCl2. (E) *In vitro* assemblies of pre-dephosphorylated GFP::CDK5RAP2(WT) and GFP::CDK5RAP2(S613E) without PLK-1 or ATP·MgCl2. Scale bars: 5 μm. (F) Quantification of results as in E. Each data point represents the mass of an individual GFP::CDK5RAP2 assembly [mean±95% c.i.; WT (*n*=1337 assemblies), S613E (*n*=2463 assemblies)]. Significant differences were assessed using a Mann–Whitney test. (G) Total number of assemblies per image from results as in E. Each data point represents the total number of assemblies per image (mean±95% c.i.; WT *n*=7, S613E *n*=7). Significant differences were assessed using a Mann–Whitney test. **P<0.01; ****P<0.0001; ns, not significant. a.u., arbitrary units.

In human cells, PLK-1 kinase activity is required to expand the amount of CDK5RAP2 at centrosomes ∼5-fold during mitosis (Haren et al., 2009; Santamaria et al., 2011). We thus tested whether PLK-1 can also regulate the assembly of our antibody-nucleated GFP::CDK5RAP2 assemblies. We incubated pre-dephosphorylated full-length GFP::CDK5RAP2 plus anti-CM2 IgM in the presence of purified kinase dead (KD) or constitutively active (CA) human PLK-1 and ATP-MgCl_2_ for 1 h at 23°C. Confocal microscopy revealed that PLK-1(CA) increased the mass of GFP::CDK5RAP2 assemblies 1.8-fold compared to the assemblies incubated with PLK-1(KD) (*P*<0.0001) (Fig. 2A,B). Moreover, we observed 1.4-fold more assemblies per image when GFP::CDK5RAP2 was incubated with PLK-1(CA) (*P*=0.0023) (Fig. 2C). This suggests our system is subject to physiological regulation and that PLK-1 kinase activity directly enhances nucleation and size of CDK5RAP2 assemblies. Our data also suggest that additional factors are required to achieve full-scale PCM growth seen in cells.

To determine which phospho-residues in CDK5RAP2 might be involved in its assembly and increased nucleation efficiency, we mapped phosphorylation sites using mass spectrometry. We identified 16 phosphorylated residues in CDK5RAP2, 56% of which fell within disordered linker regions, with the remaining 44% being in α-helical regions (Fig. 2D). These phospho-sites were found in samples containing PLK-1 but not in samples containing only pre-dephosphorylated CDK5RAP2. We also compared the phosphorylation sites found in our study to annotated phosphorylation sites of human CDK5RAP2 found in the PhosphoSite Plus (PSP) database (Hornbeck et al., 2015). Eight of the PSP-annotated residues were detected in our experiment: S255, T371, S613, S841, T1005, S1244, S1368 and S1556 (Fig. 2D, Fig. S2A,B). Of these, S613 is the only residue that has been verified to be phosphorylated by PLK-1 *in vivo* (Santamaria et al., 2011). The kinase or kinases responsible for the phosphorylation of the other previously reported residues in the PSP were unidentified; our results suggest they might be PLK-1 sites. Our experiment revealed eight new PLK-1 phosphorylation sites in CDK5RAP2: T172, T701, T712, T786, S872, T962, T1342 and S1436 (Fig. 2D). We did not observe residues known to be phosphorylated by the mitotic kinase LARRK1 (pS102 or pS140) (Hanafusa et al., 2015), indicating that our experimental conditions allow for kinase specificity.

Given that S613 was the only residue unambiguously identified to be phosphorylated by PLK-1 *in vivo* and *in vitro,* we tested whether phosphorylation of this single residue is sufficient to enhance CDK5RAP2 self-assembly *in vitro*. We purified pre-dephosphorylated GFP::CDK5RAP2 containing a glutamic acid substitution at S613 (phospho-mimetic) and compared its self-assembly capabilities to pre-dephosphorylated wild-type GFP::CDK5RAP2. The reaction contained the anti-CM2 antibody but no PLK-1 or ATP·MgCl_2_. Individual GFP::CDK5RAP2(S613E) assemblies were, on average, 1.1-fold larger than WT assemblies (*P*=0.3) (Fig. 2E,F). This result suggests that phosphorylation of residue S613 does not significantly enhance growth of CDK5RAP2 assemblies. However, we observed that S613E formed twice as many assemblies as did WT (*P*<0.0001) (Fig. 2G). This suggests that S613E is sufficient to promote CDK5RAP2 nucleation when using a synthetic nucleating seed. We also noticed more assemblies overall in this assay, which was due to the absence of MgCl_2_ in the buffer (Fig. 2G).

Together, we conclude that *in vitro* minimal centrosome scaffolds are subject to phospho-regulation by PLK-1, a key feature of *in vivo* PCM. Our results suggest that phosphorylation of CDK5RAP2 promotes its multimerization in the absence of centrioles, similar to the mechanism proposed for *C. elegans* SPD-5 (Rios et al., 2024; Woodruff et al., 2015) and *D. melanogaster* Cnn (Conduit et al., 2014). However, centrioles, other inner PCM proteins and synergistic cooperation between multiple phosphorylated residues are likely crucial for the maintenance of PLK-1 activity and for achieving maximal PCM size (Conduit et al., 2014; Rios et al., 2024; Wong et al., 2022; Woodruff et al., 2015).

### *In vitro* CDK5RAP2 micron-scale assemblies selectively recruit γ-TuRCs and nucleate microtubule asters

*In vivo*, CDK5RAP2 recruits γ-TuRCs via its NEDD1 adapter to produce robust microtubule asters, giving centrosomes their main function (Choi et al., 2010; Fong et al., 2008). However, it is not known whether CDK5RAP2 and γ-TuRCs are sufficient to achieve robust microtubule aster formation or whether additional components are required. We first used confocal microscopy to test whether CDK5RAP2 assemblies nucleate microtubules by themselves by assembling them with anti-CM2 IgM and HiLyte-647-labeled α/β-tubulin. At 37°C, GFP::CDK5RAP2 assemblies alone did not recruit α/β tubulin or generate microtubule asters (Fig. S3A).

**Fig. 3.**
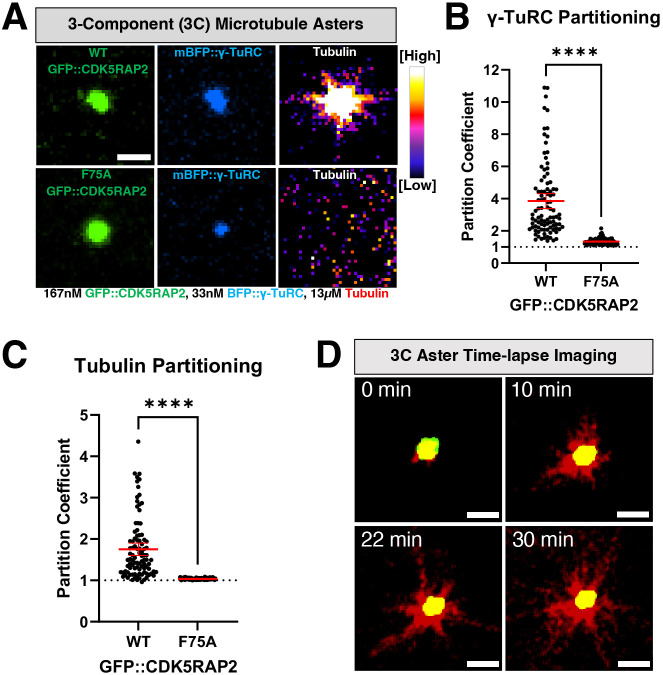
**Microtubule-nucleating activity of CDK5RAP2 scaffolds with γ-TuRCs.** (A) 3-Component (3C) reactions were assembled with purified GFP::CDK5RAP2 (WT or F75A), mBFP::γ-TuRCs, and HiLyte647-labeled α/β tubulin. Scale bar: 3 μm. (B) Quantification of γ-TuRC partitioning into GFP::CDK5RAP2 assemblies (mean±95% c.i.; WT, *n*=95; F75A, *n*=117) from results as in A. Significant differences were assessed using a Mann–Whitney test. (C) Quantification of α/β-tubulin partitioning into GFP::CDK5RAP2 assemblies (mean±95% c.i.; WT, *n*=95; F75A, *n*=117) from results as in A. Significant differences were assessed using a Mann–Whitney test. (D) Time-lapse imaging of microtubule growth from GFP::CDK5RAP2 scaffolds+mBFP::γ-TuRCs. *Z*-projections were obtained every 2 min for 30 min at 37°C. Images representative of three repeats. Scale bars: 3 μm. Error bars are sometimes too small to be visible. ****P<0.0001.

We then added purified human γ-TuRCs tagged with blue fluorescent protein (mBFP). CDK5RAP2 assemblies recruited γ-TuRCs and α/β tubulin (Fig. 3A–C). There was a strong correlation between the amount of γ-TuRC fluorescence and α/β-tubulin recruited (Pearson's correlation r=0.86; *P*<0.0001) (Fig. S3B). 90% of γ-TuRC-positive assemblies formed microtubule asters over the course of 30 min (*n*=134 assemblies) (Fig. 3A–D; Movie 1). Thus, a minimal module of self-assembled CDK5RAP2, γ-TuRCs and α/β-tubulin is sufficient to recapitulate microtubule aster formation, a key feature of centrosome function in cells.

CDK5RAP2 binds γ-TuRCs through its N-terminal CM1 domain (Choi et al., 2010; Fong et al., 2008). Mutation of phenylalanine 75 to alanine (F75A) in a truncated CDK5RAP2 construct (a.a. 51–100) disrupts γ-TuRC binding and subsequent ring closure (Choi et al., 2010; Xu et al., 2024). Whether F75 is crucial for binding or activation of γ-TuRC in the context of full-length CDK5RAP2 had not been tested. Purified CDK5RAP2(F75A) multimerized into micron-scale assemblies that poorly recruited γ-TuRCs and α/β-tubulin (Fig. 3A–C). This result reinforces the importance of F75 for the interaction between γ-TuRC and CDK5RAP2 and demonstrates that our system recapitulates binding specificity. The fact that the F75A mutant still recruits minute amounts of γ-TuRC could be an artifact of this system. Alternatively, it could suggest that additional less-important interaction motifs besides CM1 could engage with the γ-TuRC. In these mutant assemblies, there was no correlation between γ-TuRC and α/β-tubulin recruitment, indicating that the bound γ-TuRCs were not competent to bind α/β-tubulin, unlike the control case (Fig. S3C). Even in the low γ-TuRC partitioning range, GFP::CDK5RAP2(WT) scaffolds significantly recruited more α/β-tubulin than the F75 mutant (*P*<0.0001) (Fig. S3D). Consistent with this, F75A assemblies did not nucleate microtubule asters (0%, *n*=490 assemblies) (Fig. 3A). Thus, F75 is required for recruiting the γ-TuRC and improving its ability to bind α/β-tubulin.

### Kinesin HSET potentiates microtubule aster formation and induces clustering of CDK5RAP2 assemblies *in vitro*

**Fig. 4.**
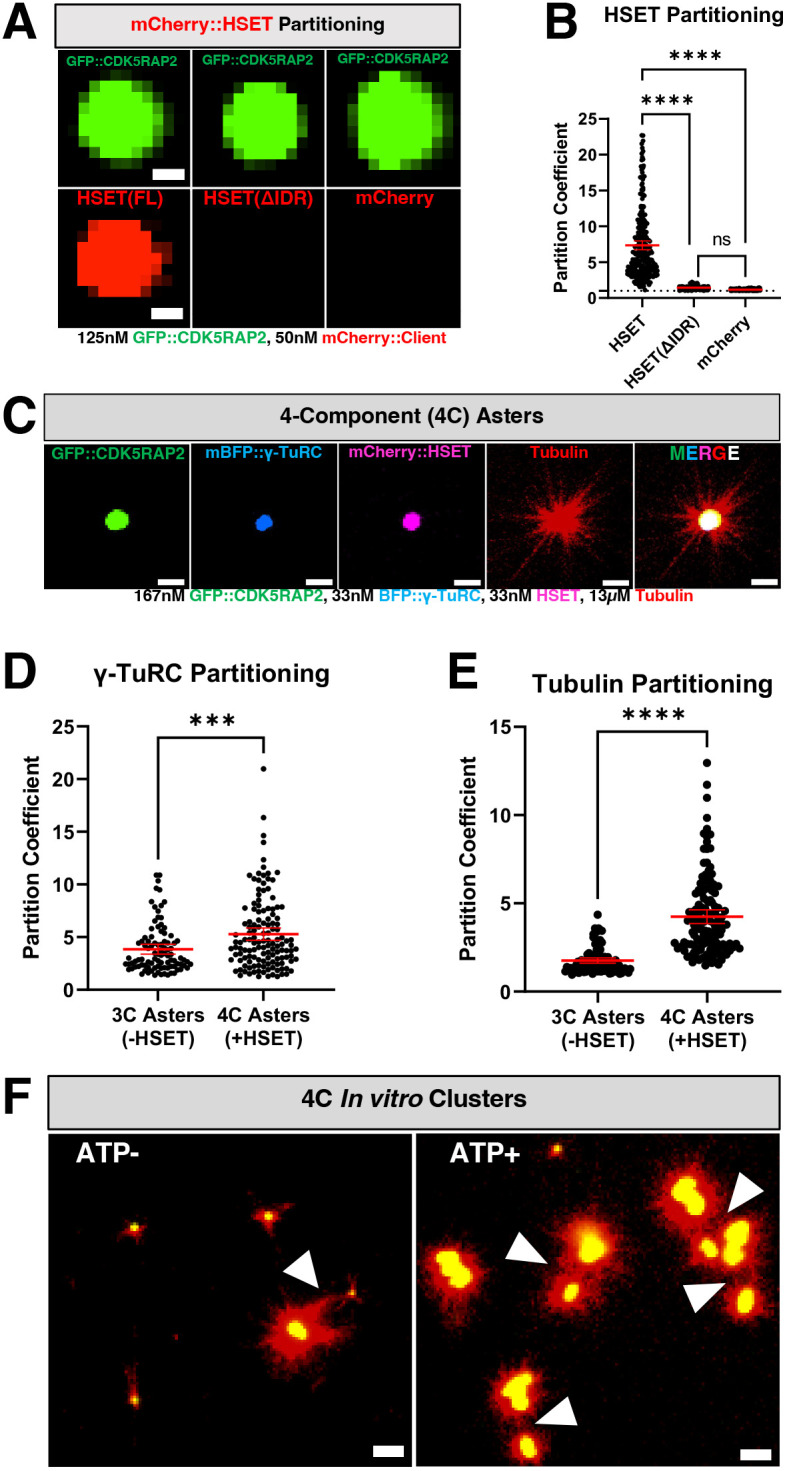
**Selective HSET recruitment, microtubule-nucleating effects and clustering of CDK5RAP2 scaffolds *in vitro*.** (A) Selective recruitment of purified mCherry::HSET (full-length), mCherry::HSET lacking its intrinsically disordered region (ΔIDR, missing a.a. 2–138) or mCherry into micron-scale GFP::CDK5RAP2 assemblies. Scale bars: 1 µm. (B) Quantification of results as in A (mean±95% c.i.; HSET(FL), *n*=293 scaffolds; HSET(ΔIDR), *n*=294 scaffolds; mCherry, *n*=285 scaffolds). Each data point represents an individual CDK5RAP2 scaffold. Significant differences were assessed using a Kruskal–Wallis test followed by Dunn's multiple comparisons test. (C) *Z*-projection of 4-Component (4C) asters consisting of 167 nM GFP::CDK5RAP2, 33 nM mBFP::γ-TuRCs, 33 nM mCherry::HSET and 13 µM HiLyte-647-labeled α/β-tubulin. Buffer consists of 50 mM KCl, 25 mM HEPES, pH7.4, IgM+. Scale bars: 3 μm. (D) Quantification of γ-TuRC partitioning in 3-component (3C) versus 4-component (4C) asters (mean±95% c.i.; 3C, *n*=95; 4C, *n*=133) from results as in C and Fig. 3A. Significant differences were assessed using a Mann–Whitney test. (E) Quantification of α/β-tubulin partitioning in 3-component (3C) vs 4-component (4C) asters. (mean±95% c.i.; 3C, *n*=95; 4C, *n*=133) from results as in C and Fig. 3A. Significant differences were assessed using a Mann–Whitney test. (F) *Z*-projection of clusters formed by 4C asters in the presence of 0 or 0.66 mM ATP plus MgCl_2_. Arrowheads indicate microtubule contacts between GFP::CDK5RAP2 scaffolds. Images representative of three repeats. Scale bars: 5 µm. Error bars are sometimes too small to be visible. ***P<0.001; ****P<0.0001; ns, not significant.

Vertebrate centrosomes stay connected to the mitotic spindle in part through the activity of HSET, a kinesin-14 motor protein that directly binds microtubules and CDK5RAP2 (Chavali et al., 2016; Mountain et al., 1999). In cancer cells with amplified centrosomes, HSET plays a crucial survival role by clustering centrosomes which prevents multipolar spindle formation (Chavali et al., 2016). We tested whether our *in vitro* system could be used as tool to investigate how HSET contributes to centrosome–spindle attachment and centrosome clustering. Thus, we first probed the ability of CDK5RAP2 to recruit HSET. We purified full-length mCherry-tagged HSET (WT), an HSET mutant that cannot bind CDK5RAP2 in cells (ΔIDR; missing a.a. 2–138; Chavali et al., 2016) and mCherry alone as a control. We then assessed their partitioning into GFP::CDK5RAP2 assemblies *in vitro*. mCherry::HSET(WT) directly interacted with GFP::CDK5RAP2 scaffolds whereas mCherry::HSET(ΔIDR) and mCherry alone did not (Fig. 4A,B). This result further demonstrates that our *in vitro* CDK5RAP2 scaffolds are specific in their recruitment of clients, recapitulating a key functional aspect of *in vivo* PCM.

Next, we asked whether *in vitro*-generated CDK5RAP2 scaffolds can bind multiple clients simultaneously and whether HSET is sufficient to cluster aster-forming CDK5RAP2 assemblies *in vitro*. Thus, we assembled GFP::CDK5RAP2 scaffolds using the anti-CM2 IgM along with human mCherry::HSET and mBFP::γ-TuRC. Then, we combined these assemblies with HiLyte647-labeled α/β-tubulin. All proteins colocalized to the micron-scale GFP::CDK5RAP2 scaffolds simultaneously to generate robust microtubule asters (Fig. 4C). We conclude that CDK5RAP2 can serve as a major centrosome scaffold for multiple clients *in vitro.*

In the presence of mCherry::HSET, GFP::CDK5RAP2 scaffolds recruited 1.4-fold more γ-TuRCs and 2.4-fold more α/β-tubulin (*P*=0.0009, *P*<0.0001, respectively) (Fig. 4D,E). More α/β-tubulin partitioned into HSET-containing CDK5RAP2 scaffolds, even when the assemblies contained similar amounts of γ-TuRCs (Fig. S4A). This effect was not due to an increase in CDK5RAP2 assembly (Fig. S4B). In the absence of γ-TuRCs, HSET recruited α/β-tubulin to scaffolds even in conditions unfavorable for tubulin polymerization (no glycerol, 23°C, no GTP) (Fig. S4C). This result is consistent with HSET's reported ability to bind soluble α/β-tubulin, likely via electrostatic interactions between the positively charged HSET tail and negatively charged clusters in tubulin (Norris et al., 2018). In conditions more favorable for polymerization (glycerol, 37°C, GTP and 20 µM tubulin), HSET–CDK5RAP2 assemblies did not make asters (Fig. S4D). We conclude that HSET concentrates α/β-tubulin into CDK5RAP2 assemblies without needing γ-TuRC or ATP, but it cannot promote aster formation in this context. We hypothesize HSET instead potentiates CDK5RAP2–γ-TuRC aster formation capabilities through increased α/β-tubulin and γ-TuRC partitioning.

Using static *Z*-projections and time-lapse recordings, we investigated whether these four-component asters could cluster together in the presence or absence of ATP·MgCl_2_ on passivated glass slides. Under these conditions, CDK5RAP2 scaffolds readily formed clusters bridged by microtubules in the presence of ATP (25 clusters, *n*=5 images), but less so in the absence of ATP (8 clusters, *n*=5 images) (Fig. 4F; Fig. S4E). Once the microtubule bridges had formed, they were stable over time (Movie 2). We did not observe complete motor-mediated constriction and fusion of CDK5RAP2 assemblies. We did not detect significant differences in area and aspect ratio of CDK5RAP2 assemblies between these conditions, suggesting that HSET motor activity does not affect CDK5RAP2 self-assembly (Fig. S4F,G). These results demonstrate that our system recapitulates HSET binding specificity and a key aspect of centrosome clustering*.* We conclude that HSET possesses two activities within CDK5RAP2 scaffolds – (1) an ATP-independent ability to concentrate tubulin and γ-TuRC, and (2) an ATP-dependent ability to cluster them via microtubules.

## DISCUSSION

Our results reveal a minimal set of components sufficient to recapitulate aspects of human PCM assembly, microtubule aster formation and ATP-dependent clustering. These components include a coiled-coil rich scaffold protein (CDK5RAP2), a microtubule-nucleating complex (γ-TuRC), an ATP-powered motor protein (HSET) and α/β-tubulin. In addition to these native proteins, specific conditions are needed to trigger scaffold assembly, including macromolecular crowding or a nucleation surface (e.g. a pentameric IgM that recruits CDK5RAP2). Specific mutations that were identified to disrupt centrosome function *in vivo* – CDK5RAP2(F75A), HSET(ΔIDR) – disrupted γ-TuRC recruitment and activation and HSET recruitment to CDK5RAP2 scaffolds *in vitro.* Finally, our system revealed a new ATP-independent role for HSET in concentrating α/β-tubulin and enhancing microtubule aster formation within the CDK5RAP2 assemblies. Thus, our reconstitution system recapitulates key features of *in vivo* PCM – including specificity of protein recruitment, regulation and activity – and reveals new insights that could not be achieved through traditional experiments in cells. Based on these results, we propose the following model for PCM assembly and function in human cells. First, the PCM scaffold assembles through multimerization of CDK5RAP2 and other proteins (e.g. Cep192 and PCNT) around a nucleation point (e.g. the centriole) using a conserved centrosome localization sequence (CM2). Second, PLK-1 phosphorylates CDK5RAP2 to potentiate its multimerization. Third, the PCM scaffold concentrates microtubule-nucleating complexes, which subsequently concentrate soluble α/β-tubulin and initiate microtubule polymerization.

Our study provides evidence that vertebrate CDK5RAP2 can multimerize on its own, and that this multimerization is enhanced by polo kinase phosphorylation, behaviors reminiscent of *C. elegans* SPD-5 (Rios et al., 2024; Woodruff et al., 2015) and *D. melanogaster* Cnn (Feng et al., 2017). Our PLK-1-independent CDK5RAP2 assemblies likely represent PCM organization in interphase, when PLK-1 is inactive but micron-scale PCM complexes can still form (Chen et al., 2022; Lawo et al., 2012). When we add active PLK-1, but not kinase dead PLK-1, CDK5RAP2 assemblies grew 1.8-fold in size. This effect was not as dramatic as natural PCM growth in cells; thus, we speculate that multiple phosphorylated residues, and likely other proteins, cooperate to achieve full-scale expansion of the human PCM scaffold. Furthermore, we saw that a single phospho-mimicking point mutation on CDK5RAP2, S613E, had no effect on GFP::CDK5RAP2 scaffold growth *in vitro* but was sufficient to enhance scaffold nucleation. Although incomplete, this simple reconstitution system nevertheless provides a strong foundation towards revealing the molecular mechanism of PLK-1-dependent centrosome expansion. Future studies should focus on dissecting this mechanism and testing whether PLK-1 phosphorylation induces conformational changes in CDK5RAP2 to promote its self-assembly, both *in vitro* and in cells.

Our experiments also shed insight into microtubule nucleation mechanisms and the minimal requirements for centrosome clustering. Previous experiments revealed that a truncated version of CDK5RAP2 (a.a. 51-100), but not an F75A mutant, was sufficient to pull down γ-TuRC in cell extracts (Choi et al., 2010). This result suggested that CDK5RAP2 recruits γ-TuRC through its CM1 domain. Whether F75 is crucial in the context of full-length CDK5RAP2 had not been tested. We saw that assemblies made with full-length, wild-type CDK5RAP2 could strongly recruit γ-TuRC whereas the F75A mutant could not. Furthermore, γ-TuRCs were only active when bound to wild-type CDK5RAP2. These data indicate that CM1 is the primary mediator of γ-TuRC recruitment and that F75 is crucial for activating γ-TuRCs, likely due to mediating ring closure (Xu et al., 2024). Our data also show that CDK5RAP2 multimerization is a key step in microtubule aster formation. We speculate that aster formation might further be improved by components that help γ-TuRC binding, for example through relieving auto-inhibitory folding that could obscure the CM1 domain (Yang et al., 2023).

Addition of the kinesin-14 motor HSET improved microtubule aster formation by CDK5RAP2–γ-TuRC assemblies. However, the mechanism of this synergy is not fully clear. In the presence of soluble α/β-tubulin and without CDK5RAP2, HSET can form microtubule asters using its N-terminal IDR and C-terminal motor domain (Hentrich and Surrey, 2010; Norris et al., 2018; Roostalu et al., 2018). Within CDK5RAP2 scaffolds, we found that HSET by itself could recruit α/β-tubulin but could not nucleate microtubules, even when using similar protein concentrations as previously described (Norris et al., 2018). What could explain these differences? We speculate that the intrinsic microtubule nucleation capabilities of HSET are hindered by its interaction with the CDK5RAP2 scaffold. HSET also improved γ-TuRC recruitment into CDK5RAP2 scaffolds. Thus, HSET likely promotes microtubule polymerization within the PCM by locally concentrating soluble α/β-tubulin and γ-TuRC in an ATP-independent manner. Adding ATP promoted clustering of the CDK5RAP2–γ-TuRC–HSET assemblies, consistent with a reported role for HSET motor activity in clustering multiple centrosomes in cells (Chavali et al., 2016). Based on these observations, we propose that HSET drives centrosome clustering by promoting microtubule polymerization through concentration of soluble tubulin and crosslinking microtubules.

*In vitro* reconstitution systems have been instrumental in elucidating mechanisms of centrosome assembly and function in invertebrates (Conduit et al., 2014; Feng et al., 2017; Woodruff et al., 2017, 2015), but they have been lacking for human cells. Moreover, key deficiencies exist with extant reconstitution systems. Our own *C. elegans* system recapitulated PCM scaffold assembly and microtubule aster formation, but it relied on non-natural crowding agents to induce scaffold assembly and lacked the most prominent microtubule nucleator, the γ-TuRC (Woodruff et al., 2017). The *D. melanogaster* system recapitulated Polo kinase-regulated formation of a sparse Cnn scaffold but has not yet achieved a condensed, microtubule aster-forming state (Feng et al., 2017). Our current work improves upon these deficiencies using full-length human CDK5RAP2, a centriole-mimicking nucleator, the complete γ-TuRC, other pure proteins and no crowding agents. This human system recapitulates important centrosome behaviors, such as PCM scaffold nucleation, PCM expansion, phospho-regulation, microtubule nucleation and client selectivity. Combined with previous studies, our work reveals that PCM in diverse species assembles through a universal mechanism – Polo kinase-regulated multimerization of a supramolecular scaffold that concentrates client proteins.

Our work thus provides a versatile tool for studying CDK5RAP2 function and its regulation in isolation from cellular complexity. In the future, this system could be improved with: (1) more robust phospho-regulation of CDK5RAP2 scaffold expansion, perhaps via inclusion of different kinases and phosphatases; (2) inclusion of other potential scaffold proteins such as Cep192 and pericentrin; and (3) inclusion of other effector molecules, such as microtubule nucleating or capping complexes. Nevertheless, in its current state, our system has revealed new mechanistic insight into human centrosome biology and holds promise for investigating centrosome dysfunction in human disease.

## MATERIALS AND METHODS

All reagents are available upon request by contacting the corresponding author.

### Experimental model and cell line details

For the expression of recombinant proteins (listed in Table S1), we used SF9-ESF *Spodoptera frugiperda* insect cells (Expression Systems) grown at 27°C in ESF 921 Insect Cell Culture Medium (Expression Systems) supplemented with fetal bovine serum (2% final concentration). DLD-1 cells expressing CDK5RAP2-2xmNeonGreen were a gift from Andrew Holland (Johns Hopkins University, Baltimore, MD, USA) and were derived from the parental cell line available at ATCC (CCL-221).

### Protein purification

All expression plasmids are listed in Table S1. Full-length human GFP::CDKRAP2 proteins (WT, F75A and ΔCM2), human PLK-1 (constitutively active and kinase dead) and mCherry::HSET constructs (full-length, ΔIDR) were inserted into a baculoviral expression plasmid (pOCC7, pOCC29 or pOCC195) using standard restriction cloning. Baculoviruses were generated using the FlexiBAC system (Lemaitre et al., 2019) in SF9 cells. Protein was harvested 72 h after infection during the P3 production phase. Cells were collected (300 ***g*** for 10 min), washed and resuspended in harvest buffer (25 mM HEPES, pH 7.4, 150 mM NaCl). All subsequent steps were performed at 4°C. Cell pellets were resuspended in Buffer A (25 mM HEPES, pH 7.4, 30 mM imidazole, 500 mM KCl, 0.5 mM DTT, 1% glycerol, 0.1% CHAPS) plus protease inhibitors and then lysed using a dounce homogenizer. Proteins were bound to Ni-NTA (Qiagen), washed with 10 column volumes of Buffer A and eluted with 250 mM imidazole. For CDK5RAP2, cell lysates were passed over maltose-binding protein (MBP) selector beads (NanoTag Biotechnologies) and washed with five column volumes of Buffer C (25 mM HEPES, pH 7.4, 500 mM NaCl, 0.5 mM DTT, 1% glycerol and 0.1% CHAPS), then eluted by performing overnight cleavage with PreScission protease, and then passing over Ni-NTA beads to remove the Precission protease. Eluted proteins were concentrated using 3–100 K MWCO Amicon concentrators (Millipore). All proteins were aliquoted in PCR tubes, flash-frozen in liquid nitrogen, and stored at −80°C. Protein concentration was determined by measuring absorbance at 280 nm using a NanoDrop ND-1000 spectrophotometer (Thermo Fisher Scientific). To dephosphorylate CDK5RAP2 proteins, MBP-trap bound GFP::CDK5RAP2 was incubated for 1 h at room temperature in dephosphorylation buffer [1× PMP buffer (NEB), 1 mM MnCl_2_ and 40,000 U lambda phosphatase (400,000 U/ml, NEB]. Beads were then washed twice with buffer C at 4°C during the regular washing steps (total of seven washes) to remove lambda phosphatase. Dephosphorylation of GFP::CDK5RAP2 was confirmed by PTM identification using mass spectrometry showing effective removal of 99.9–100% of phosphates.

Human γTuRCs were purified as previously described (Brito et al., 2024). In short, HeLa-Kyoto cells expressing mBFP::γTuRCs were resuspended in lysis buffer (50 mM HEPES, 150 mM KCl, 5 mM MgCl2, 1 mM EGTA, 1 mM DTT, 0.1 mM GTP, pH 7.4) containing protease inhibitors and DNase I (10 μg/ml, Sigma-Aldrich). Resuspended cells were lysed, clarified twice by centrifugation (17,000 ***g***, 15 min, 4°C) and filtered. Lysates were desalted, supplemented with protease inhibitors and loaded onto a 1 ml HiTrap SP Sepharose FF column connected in tandem with 1 ml streptavidin mutein matrix beads (Sigma-Aldrich) packed into a Tricorn 5/50 column (GE-Healthcare). The streptavidin mutein matrix column was washed with 30 ml storage buffer, 30 ml wash buffer [lysis buffer containing 200 mM KCl and 0.2% (v/v) Brij-35] and 30 ml storage buffer. Proteins were eluted with storage buffer supplemented with 5 mM D-biotin. The buffer was then exchanged into storage buffer, centrifuged (17,000 ***g***, 10 min, 4°C) and separated by size exclusion chromatography using a Superose 6 10/300 GL column. γTuRC peak fractions were pooled, concentrated, ultracentrifuged (TLA 100 rotor, 80,000 rpm, 10 min, 4°C), snap frozen and stored in liquid nitrogen. Porcine tubulin was used in this study and was purified as previously described (Gell et al., 2011).

### *In vitro* assembly of CDK5RAP2 scaffolds

Purified GFP::CDK5RAP2 stocks were dialyzed prior to their use in reaction buffer (50 mM KCl, 25 mM HEPES, pH7.0). Dialysis took place overnight at 4°C using 10 K MWCO MINI Dialysis Units (Thermo Fisher Scientific). Assembly reactions were carried on in 10 μl volumes at room temperature for 1–2 min. Final protein concentrations and buffer conditions were 0–120 mM GFP::CDK5RAP2, 150 mM KCl, 25 mM HEPES, pH7.4 for crowder-based assemblies and 125 mM GFP::CDK5RAP2, 50 mM KCl, 25 mM HEPES, pH7.4 for antibody-based assemblies. Scaffolds were assembled either using crowding agents [PEG (1–10% w/v), PVP (10% w/v), Ficoll (10% w/v), Dextran (10% w/v) or Lysozyme (10% w/v)] or monoclonal mouse antibodies (IgM) raised against the human CDK5RAP2 CM2 domain (Invitrogen, Reference ID: MA5-37609, LOT ID: YD3903101). The commercial antibody stock (5–10 mg/ml) was diluted 1:100 in reaction buffer prior to its use. To induce CDK5RAP2 assembly using anti-CDK5RAP2 IgMs, 1 μl of the diluted antibody stock was added to 9 μl of the assembly reaction containing GFP::CDK5RAP2 diluted in reaction buffer. Final antibody concentration was 5-10 μg/ml (5-11pM). Assembly of pre-dephosphorylated WT and S613E GFP::CDK5RAP2 scaffolds as well as reactions containing mCherry or mCherry::HSET were also induced using antibodies (120 nM GFP::CDK5RAP2 final concentration). Control mCherry, full-length and ΔIDR mCherry::HSET stocks were diluted using 25 mM HEPES pH 7.0 prior to their addition to match salt concentrations in reaction buffer. Final concentrations of these proteins were 50 nM (10 μl volume).

### Reversibility assays

For both protein dilution and salt increase reversibility assays, GFP::CDK5RAP2 assemblies were generated using an anti-CM2 IgM in plastic PCR tubes (20 μl reaction volumes). After 5 min, these were transferred to a 96-well plate Corning imaging plate (ref. 4580) then mounted on a Nikon Eclipse Ti2 Microscope for imaging. Pictures ‘before’ addition of salts or diluting buffer were taken using a 60×1.2 NA Plan Apochromat water-immersion objective. 488-nm excitation (15% laser power) was recorded using 2×2 binning. For protein dilution, 20 μl of 50 mM KCl, 25 mM HEPES, pH 7.4 buffer was added. For salts, 20 μl of a 500 mM KCl, 25 mM HEPES, pH7.4 containing 125 μM GFP::CDK5RAP2 was added. ‘After’ images were taken within 5 min after addition using the exact same imaging settings.

### Photobleaching of CDK5RAP2 assemblies

GFP::CDK5RAP2 assemblies were generated exactly as described above. However, only 10 μl were transferred onto a 22×22 microscope coverslips through gentle pipetting for this assay. Samples were sandwiched between a coverslip over and a pre-cleaned 75×25×1 mm microscope slide (VWR Vistavision) and sealed with non-toxic transparent nail polish, then transferred onto a Nikon Eclipse microscope and imaged using a 100×1.35 NA silicone objective using the same imaging settings as described above. A single pre-photobleaching *Z*-stack image was acquired prior to photobleaching. The same GFP::CDK5RAP2 assemblies were then photobleached by shining a 405 nm laser (500 ms, 50% laser power) over a circular region of interest that partially or fully covered any given assembly. Control photobleaching events took place away from GFP::CDK5RAP2 assemblies. Post-photobleaching *Z*-stacks were acquired every 10 s for 160 s.

### PLK-1 phosphorylation reactions and CDK5RAP2 phospho-mapping

PLK-1-driven expansion of GFP::CDK5RAP2 assemblies *in vitro* was achieved by incubating pre-dephosphorylated GFP::CDK5RAP2 with purified constitutively active (CA) human PLK-1 (T194D). The reaction took place at room temperature for 1 h in reaction buffer supplemented with 20× ATP·MgCl2 (final concentration, 0.5 mM). As a control, we incubated CDK5RAP2 with purified kinase dead (KD) human PLK-1 (K82A) under the same conditions. Final concentrations were 120 μM GFP::CDK5RAP2, 50 nM PLK-1 (KD/CA), 200 μM ATP, 5 mM MgCl2, 50 mM KCl, 25 mM HEPES, pH 7.0 in 10 μl total volume. To identify PLK-1 phosphorylation sites in CDK5RAP2, 4.00 µM of pre-dephosphorylated unlabeled CDK5RAP2 was incubated with 1 µM of active human PLK-1 (MedChem Express), 1 mM ATP, 25 mM MgCl2 for 1 h at room temperature in 150 mM KCl, 25 mM HEPES, pH 7.4 (10 µl total volume). Reactions are stopped by addition of 2× SDS loading sample buffer, ran on a 4–20% protean TGX gel for 35 min at 200 V, and stained with the Coomassie-based stain InstantBlue. CDK5RAP2 bands located at the height of 215 kDa were excised under sterile conditions and sent for phosphorylation PTMID. Three other CDK5RAP2 samples with no PLK-1 were submitted as non-phosphorylated controls. Samples were digested overnight at 37°C with trypsin, chymotrypsin and Glu-C (Sigma) and results were combined to maximize protein coverage (>80%). Positive phosphorylated residues were those found in the PLK-1 plus ATP·MgCl_2_ condition and not in control reactions.

### *In vitro* aster formation

Aster assembly began by preparing a 1:5 labeled to unlabeled porcine tubulin mix on ice. To do this, we combined two tubes of lyophilized 100 μM commercial porcine tubulin (Cytoskeleton, Inc.; TL670M-A) labeled with HiLyte647 dissolved by adding 2.88 μl of ultra-pure deionized water to each tube (5.76 μl total). Labeled tubulin was then combined with 10.62 μl of 137 μM unlabeled porcine tubulin and 1.82 μl of 100 mM GTP. Final mix consists of 100 μM tubulin, 10 mM GTP. This mix is centrifuged (80,000 rpm for 20 min at 4°C) using an Optima MAX-XP ultracentrifuge in a TLA100 rotor. The top 14 µl of the mix was transferred to a PCR tube on ice for use without disrupting precipitated tubulin. Tubulin polymerization aswis assessed prior to aster formation by combining 2 μl of the spun tubulin mix with 1.5 μl of 50% glycerol (w/v), 1 μl 5× BRB80, 4 mM GTP and 0.5 μl 10 mM DTT. Final 1× BRB80 contains: 80 mM PIPES, 1 mM MgCl2, 1 mM EGTA, pH 6.8 with KOH. Under these conditions, numerous microtubules should spontaneously polymerize and can be visualized using a fluorescent microscope. In parallel, GFP::CDK5RAP2 scaffolds were prepared by combining pre-dialyzed GFP::CDK5RAP2 (WT or F75A) (1 μM final) in reaction buffer (50 mM KCl, 25 mM HEPES, pH7.4), 1 μl of 1:100 anti-CDK5RAP2 IgMs diluted in reaction buffer (6.25–12.5 μg/ml final), 2 μl of 200 nM mBFP::γTuRCs in reaction buffer (50 nM final), and reaction buffer up to 8 µl in PCR tubes at room temperature. We can incorporate HSET into the reaction for the clustering assays by replacing 0.4 μl of reaction buffer with 8 μM mCherry::HSET in reaction buffer (0.4 μM final). Aster reactions were also assembled in PCR tubes on ice by combining 1.5 μl of 50% glycerol (w/v), 1 μl 5XBRB80, 0.5 μl of 10 mM DTT, 1.2 μl of pre-assembled CDK5RAP2 scaffolds plus γTuRCs (with or without HSET), 0.8 μl of spun tubulin mix (13 µM final) and 1 μl of 20× ATP·MgCl2 (666 μM final ATP, 16.6 mM MgCl2). The ATP added was pre-mixed with catalase (0.1 mg/ml final; Sigma; C40) and glucose oxidase (1 mg/ml final; Biophoretics; 22778.01). The final GTP concentration was 1.3 mM and glycerol was 12.5% (w/v). All samples were then immediately transferred to a glass slide and imaged at 37°C using a TokaiHit incubation chamber. In reactions where ATP·MgCl2 was not required, equal amounts of water were added instead.

### Recruitment of soluble tubulin to CDK5RAP2 scaffolds by HSET

A 100 µM tubulin mixture was prepared without GTP (1:5 HiLyte647 labeled tubulin to unlabeled tubulin). To ensure removal of aggregates, the tubulin mix was spun at 80,000 rpm for 20 min at 4°C using an Optima MAX-XP ultracentrifuge in a TLA100 rotor. Only the top 3 µl of the spun tubulin mix was used for the assays and kept on ice. 1 µl of the tubulin mix was combined with 3.5 µl of pre-assembled GFP::CDK5RAP2 and mCherry::HSET assemblies (581 nM and 593 nM final concentrations respectively), 1 µl of 20× ATP·MgCl2 (0.666 nM ATP,16.65 mM MgCl_2_ final concentration), and 0.5 µl of 10 mM DTT (833 nM final concentration; 6 µl final volume). No glycerol was added to the mix. Tubulin concentration into CDK5RAP2 assemblies was assessed by capturing far-red signal (647 nm) taking 41 0.5 µm *Z*-stacks using a 100×1.35 NA silicone objective confocal microscope at 23°C; 488-nm excitation (15% laser power, 100 ms exposure) followed by 561 nm (10% laser power, 100 ms exposure) and 647 nm (10% laser power, 300 ms exposure) was used. Images were captured using 2×2 binning.

### Assessing HSET and CDK5RAP2-mediated aster formation

Aster reactions were assembled the same way as described in the ‘*In vitro* aster formation’ section. HSET and ATP were included in the mix but addition of γTuRCs was replaced by buffer. Tubulin concentrations were increased to 20 μM and samples were imaged at 37°C. *Z*-stack images were captured using a 100× silicon objective.

### Microscopy of CDK5RAP2 scaffolds and *in vitro* asters

Upon assembly in PCR tubes, scaffold reactions and aster reactions were transferred to 22×22 mm microscope cover slips through gentle pipetting. Samples were then sandwiched by placing the coverslip over pre-cleaned 75×25×1 mm microscope slides (VWR Vistavision) and sealed with non-toxic transparent nail polish. These were then immediately transferred to a TokaiHit incubation chamber set at 37°C mounted on a Nikon Eclipse Ti2 microscope for imaging. For clustering assays, we passivated both the coverslip and slide with mPeg-Silane the day prior imaging using a standard passivation protocol. Time-lapse images were acquired with a Yokogawa confocal scanner unit (CSU-W1), piezo Z stage, and an iXon Ultra 888 EMCCD camera (Andor), controlled by Nikon Elements software. We used a 60×1.2 NA Plan Apochromat water-immersion objective or 100×1.35 NA silicon objective to acquire 41 0.5-µm Z-stacks with 100 ms exposures every 2 min for 30 min; 488-nm excitation (15% laser power) was recorded using 2×2 binning followed by 405 nm excitation (10% laser power, 2×2 binning), 561 nm excitation (10% laser power, 2×2 binning) and 647 nm excitation (10% laser power, 1×1 binning) was used. Iris intensity was set at 92.3%.

### Image quantification and statistical analysis

Images were analyzed using semiautomated, threshold-based particle analysis in FIJI software. Data were plotted and statistical tests were performed using GraphPad prism. The sample size, measurement type, error type and statistical test are described in the figure legends where appropriate. Statistical significance is defined as: *P<0.05, **P<0.01, ***P<0.001 ****P<0.0001, and ns, not significant. Integrated fluorescence intensity is defined as area of an assembly multiplied by its mean fluorescence intensity. This value is displayed in arbitrary units (a. u.). Partition coefficient (PC) is measured by (1) identifying CDK5RAP2 scaffolds using a threshold-based system, (2) assessing standard deviation above background, and (3) measuring the mean fluorescence intensity of mBFP, mCherry, HiLyte-647 or Alexa Fluor inside any given assembly after camera noise subtraction, and (4) dividing that mean fluorescence intensity by background mean fluorescence intensity after camera noise subtraction. PC<1 indicates exclusion from GFP::CDK5RAP2 assemblies. PC=1 indicates no selective enrichment or exclusion. PC>1 indicates selective enrichment within GFP::CDK5RAP2 assemblies. Aspect ratio is defined as the ratio between the longest and shortest diameter of a given assembly. Perfectly circular objects will have an aspect ratio of 1 whereas elongated objects (such as rectangles) will have aspect ratios >1.

## Supplementary Material



10.1242/joces.264121_sup1 Supplementary information
