# Supplementary material for: In vitro reconstitution of a minimal human centrosome scaffold capable of forming and clustering microtubule asters
Source: J Cell Sci. 2025 Jun 27;138(12):jcs264121. doi: 10.1242/jcs.264121 (PMC12273628; doi:10.1242/jcs.264121)
Supplement: Supplementary information [file joces-138-264121-s1.pdf]

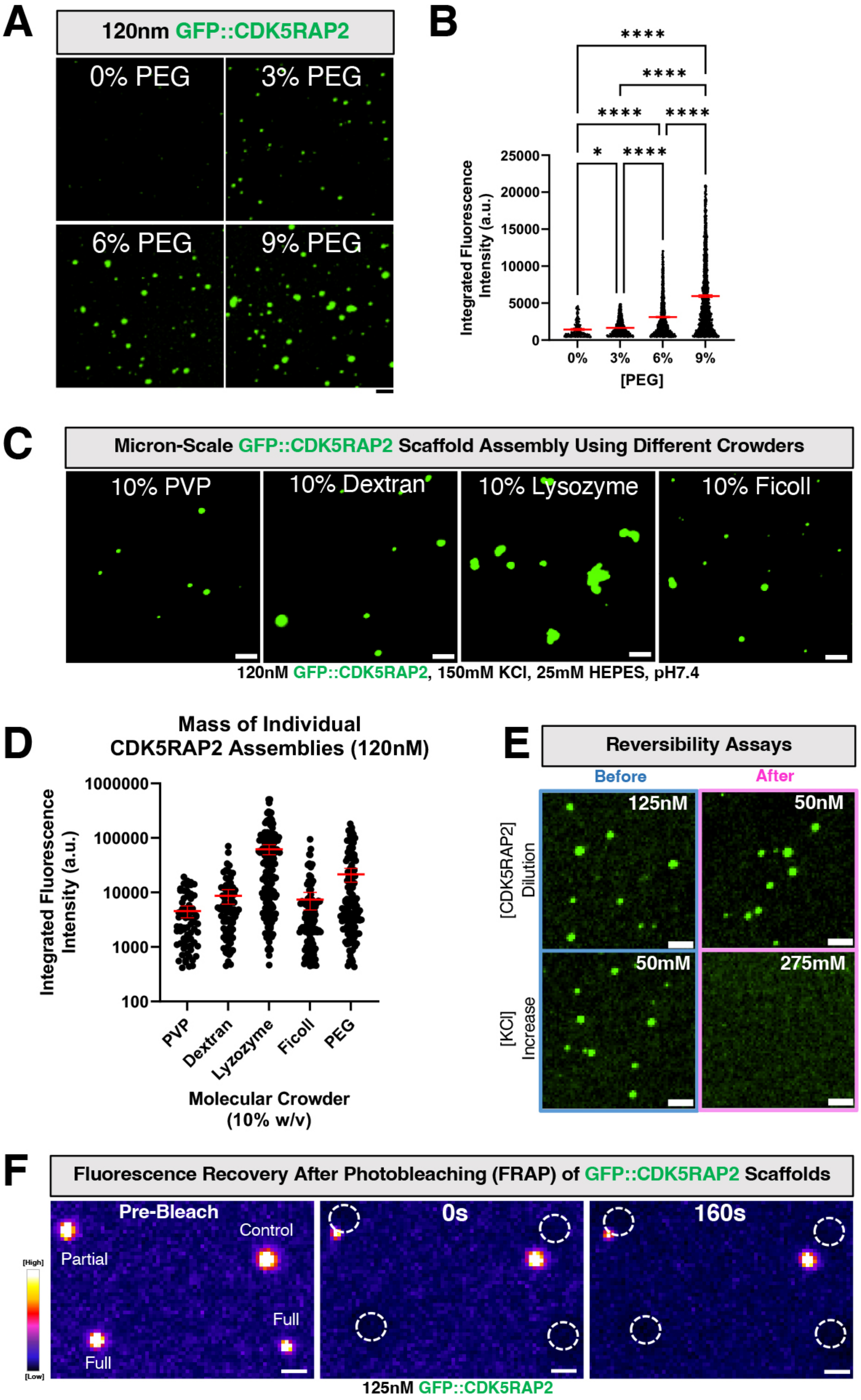
A
120nm GFP::CDK5RAP2
0% PEG
3% PEG
6% PEG
9% PEG
B
Integrated Fluorescence Intensity (a.u.)
25000
20000
15000
10000
5000
0
0%
3%
6%
9%
[PEG]
****
*
C
Micron-Scale GFP::CDK5RAP2 Scaffold Assembly Using Different Crowders
10% PVP
10% Dextran
10% Lysozyme
10% Ficoll
120nM GFP::CDK5RAP2, 150mM KCl, 25mM HEPES, pH7.4
D
Mass of Individual CDK5RAP2 Assemblies (120nM)
Integrated Fluorescence Intensity (a.u.)
1000000
100000
10000
1000
100
PVP
Dextran
Lyzozyme
Ficoll
PEG
Molecular Crowder (10% w/v)
E
Reversibility Assays
Before
After
[CDK5RAP2] Dilution
125nM
50nM
[KCl] Increase
50mM
275mM
F
Fluorescence Recovery After Photobleaching (FRAP) of GFP::CDK5RAP2 Scaffolds
Pre-Bleach
Control
Partial
Full
Full
0s
160s
[High]
[Low]
125nM GFP::CDK5RAP2

Fig. S1. Assembly of CDK5RAP2 scaffolds using different PEG concentrations and different crowders.

A) 120nM purified GFP::CDK5RAP2 combined with various PEG concentrations (0%, 3%, 6%, 9%)(w/v). Scale bar, 5µm.

B) Quantification of panel 1B. Significant differences were assessed using One-way ANOVA followed by Tukey's multiple comparisons test. Y-axis represents integrated fluorescence intensity of CDK5RAP2 assemblies generated at various PEG concentrations.

C) Micron scale assemblies of GFP::CDK5RAP2 generated using 10% (w/v) PVP, Dextran, Lyzozyme or Ficoll. Scale bar, 5µm.

D) Quantification of panel 1C (mean +/- 95% C.I.; PVP (n=69 assemblies), Dextran (n=79 assemblies), Lyzozyme (n=173 assemblies), Ficoll (n=105 assemblies), PEG (n=130 assemblies)). Y-axis represents integrated fluorescence intensity (area x mean intensity) of individual GFP::CDK5RAP2 assemblies represented in Log10 scale.

E) Protein dilution and salt-based reversibility assays of GFP::CDK5RAP2 assemblies. Scale bar, 5µm.

F) Fluorescence recovery after photobleaching (FRAP) of GFP::CDK5RAP2 assemblies. Circles indicate photobleached region. Scale bar 3µm.

Journal of Cell Science • Supplementary information

A

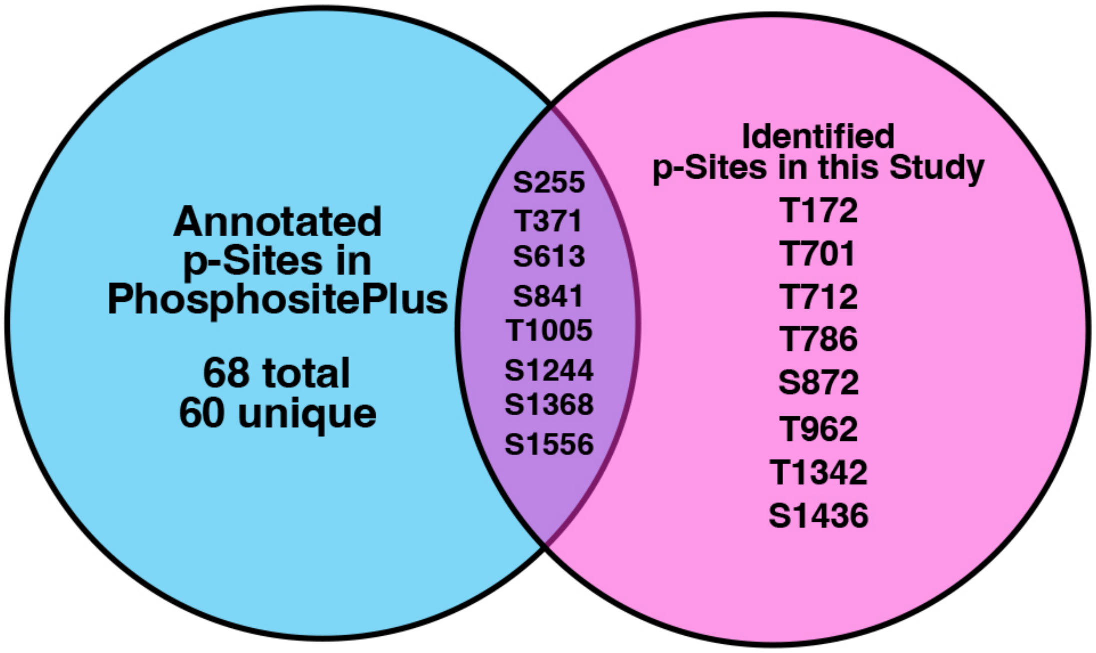

B

Annotated CDK5RAP2 p-Sites on PhosphoSite Plus

102	371*	547	732	1020	1238	1428
128	374	548	791	1061	1244*	1488
140	391	550	815	1074	1343	1490
161	392	564	841*	1076	1349	1548
255*	400	576	945	1077	1350	1556*
274	461	613*	947	1102	1354	1617
324	463	682	952	1111	1360	1666
360	466	697	1001	1164	1362	1885
366	468	698	1005*	1172	1368*	
369	486	706	1017	1173	1389	

*Annotated p-sites also found in our study

Fig. S2. CDK5RAP2 phospho-site information

A) Venn diagram indicates shared and unique p-Sites in CDK5RAP2 reported in the phospho-proteome database PhosphoSite Plus (PSP)(Blue), the phospho-sites found in our study (Pink) and the phospho-sites found in both (Purple).

B) List of annotated phospho-sites in PhosphoSite Plus (Hornbeck et al., 2015). Annotated residues also found in our study are in purple.

Journal of Cell Science • Supplementary information

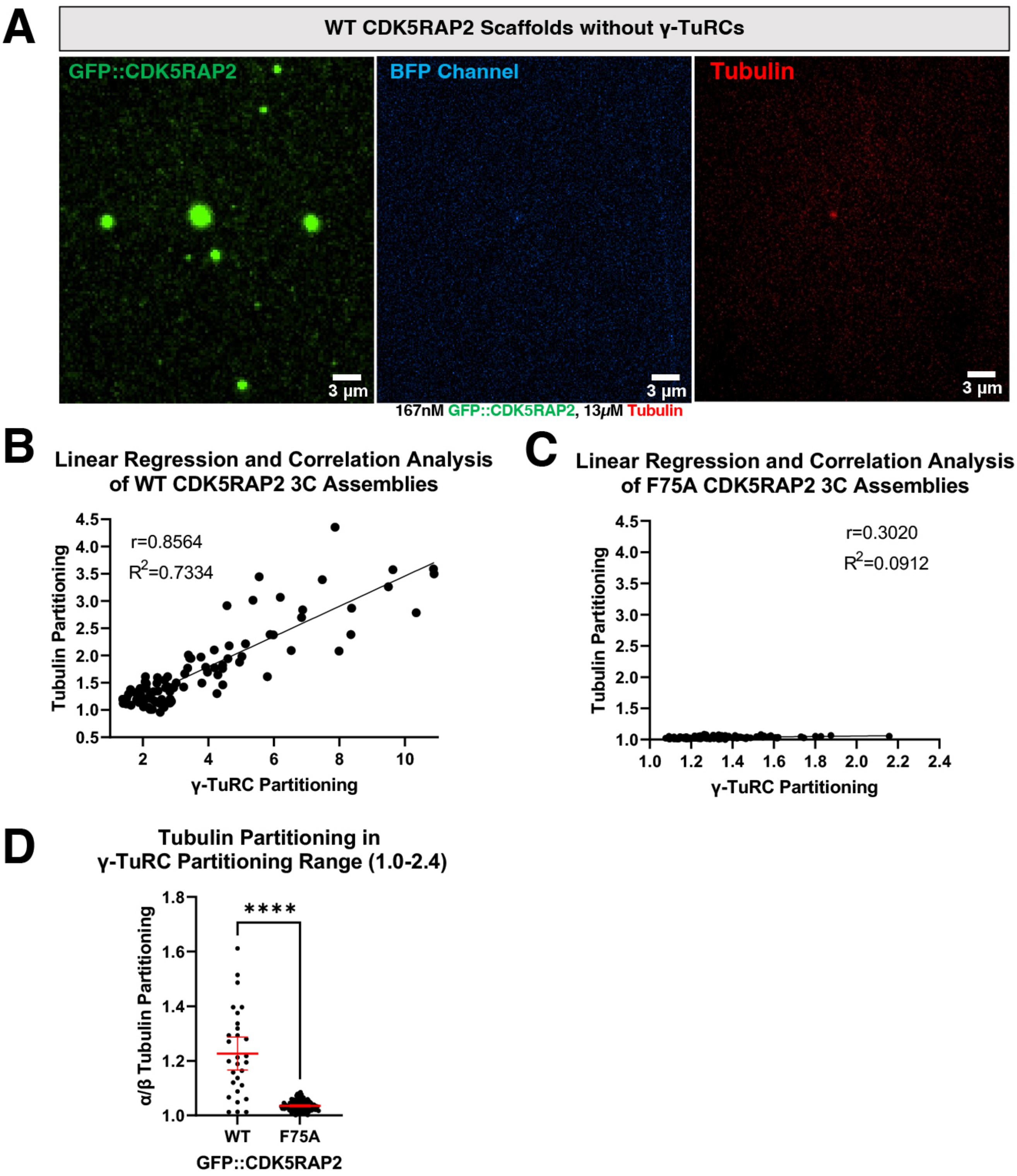

A
WT CDK5RAP2 Scaffolds without γ-TuRCs
GFP::CDK5RAP2
BFP Channel
Tubulin
3 μm
167nM GFP::CDK5RAP2, 13μM Tubulin
B
Linear Regression and Correlation Analysis of WT CDK5RAP2 3C Assemblies
r=0.8564
R2=0.7334
Tubulin Partitioning
γ-TuRC Partitioning
C
Linear Regression and Correlation Analysis of F75A CDK5RAP2 3C Assemblies
r=0.3020
R2=0.0912
Tubulin Partitioning
γ-TuRC Partitioning
D
Tubulin Partitioning in γ-TuRC Partitioning Range (1.0-2.4)
****
α/β Tubulin Partitioning
WT
F75A
GFP::CDK5RAP2

Fig. S3. Regression analysis of 3C asters and microtubule nucleation capabilities of CDK5RAP2 alone

A) 167nM GFP::CDK5RAP2(WT) scaffolds assembled using anti-CM2 IgMs in the presence of 13µM Hi-Lyte-labeled α/β tubulin mix (25mM HEPES, 50mM KCl, pH7.4).

B) Linear regression and correlation analysis of CDK5RAP2(WT) assemblies. γ-TuRC partitioning (X-axis) is plotted against α/β tubulin partitioning (Y-axis) within any given CDK5RAP2 assembly. Each data point represents a CDK5RAP2 scaffold (n=95).

C) Linear regression and correlation analysis of CDK5RAP2(F75A) assemblies. γ-TuRC partitioning (X-axis) is plotted against α/β tubulin partitioning (Y-axis) within any given CDK5RAP2 assembly. Each data point represents a CDK5RAP2 scaffold (n=117).

D) Comparison of tubulin partitioning within a specified γ-TuRC partitioning range (1.0-2.4). Each data point represents a CDK5RAP2 scaffold (mean +/- 95% C.I.; WT n=29, F75 n=117). Differences were assessed using a Mann-Whitney test.

Journal of Cell Science • Supplementary information

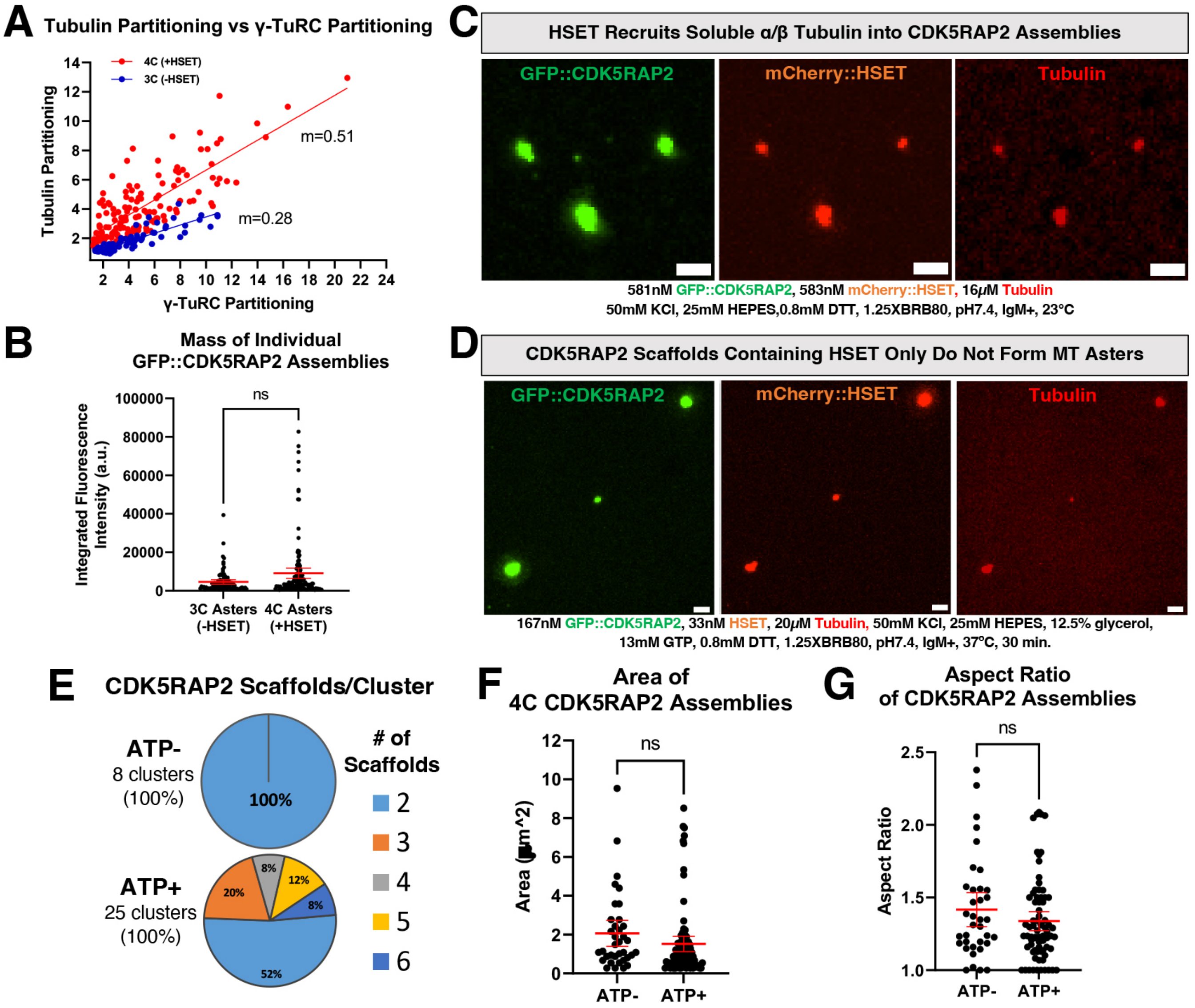

Fig. S4. Additional analyses of CDK5RAP2 assemblies containing HSET

A) Regression analysis of 4C (+HSET) vs 3C (-HSET) asters. γ-TuRC partitioning (X-axis) is plotted against α/β tubulin partitioning (Y-axis). Each data point represents a GFP::CDK5RAP2 scaffold (3C, n=95; 4C, n=133).

B) 3C vs 4C integrated fluorescence intensity of GFP::CDK5RAP2 scaffolds. Each data point represents a GFP::CDK5RAP2 scaffold (3C, n=88; 4C, n=133). Significant differences were assessed using a Mann-Whitney test.

Journal of Cell Science • Supplementary information

C) mCherry::HSET can recruit α/β tubulin to GFP::CDK5RAP2 assemblies in the absence of GTP and glycerol at 23°C. Reactions contain 581nM GFP-CDK5RAP2, 583nM mcherry-HSET, 16μM Hi-Lyte-labeled α/β tubulin mix, anti-CM2 IgM. Buffer conditions are: 25mM HEPES, 50mM KCl, 666nM ATP, 16.65mM $MgCl_2$ pH7.4. Scale bar, 3μm.

D) GFP::CDK5RAP2 scaffolds containing mCherry-HSET only do not nucleate microtubule asters. This assay is done in the presence of 20μM Hi-Lyte-labeled α/β tubulin mix, anti-CM2 IgM at 37°C. Buffer conditions are the same as figure 4C. Scale bar, 5μm.

E) Quantification of clusters and CDK5RAP2 scaffolds per cluster in ATP+/- conditions. Clusters are defined as local collections of CDK5RAP2 scaffolds connected via microtubule asters. ATP- (8 clusters from 5 images), ATP+ (25 clusters from 5 images).

F) Area of GFP fluorescence per 4C assembly (mean +/- 95% C.I.; ATP, n=36 assemblies; ATP+, n=87 assemblies). Significant differences were assessed using a Mann-Whitney test.

G) Aspect ratio of 4C scaffolds (mean +/- 95% C.I.; ATP, n=36 assemblies; ATP+, n=87 assemblies). Significant differences were assessed using a Mann-Whitney test.

Journal of Cell Science • Supplementary information

Table S1.

PROTEIN	JWV	pOCC Vector	Purification Tags (PTs)	Fluorescent Tags
CEP215/CDK5RAP2 (FL)	92	pOCC29	N-term MBP-PreScission, C-term PreScission-6xHis	N-term eGFP
CEP215/CDK5RAP2 (F75A)	145	pOCC29	N-term MBP-PreScission, C-term PreScission-6xHis	N-term eGFP
CEP215/CDK5RAP2 ΔCM2 (Δ1715-1814)	154	pOCC29	N-term MBP-PreScission, C-term PreScission-6xHis	N-term eGFP
HSET	151	pOCC195	C-term PreScission-6xHis	C-term mCherry
HSET(ΔIDR) (Δ2-150)	152	pOCC195	C-term PreScission-6xHis	C-term mCherry
PLK-1(T210D)	142	pOCC7	C-term PreScission-6xHis	None
KD PLK-1 (K82A)	144	pOCC7	C-term PreScission-6xHis	None
**PROTEIN**	**JWB**	**Plasmid Name/Number**	**Purification Tags (PTs)**	**Fluorescent Tags**
mCherry	68	pET AviTag His6 mCherry LIC cloning vector, Plasmid #29722	N-term 6xHis-TEV	Itself

Journal of Cell Science • Supplementary information

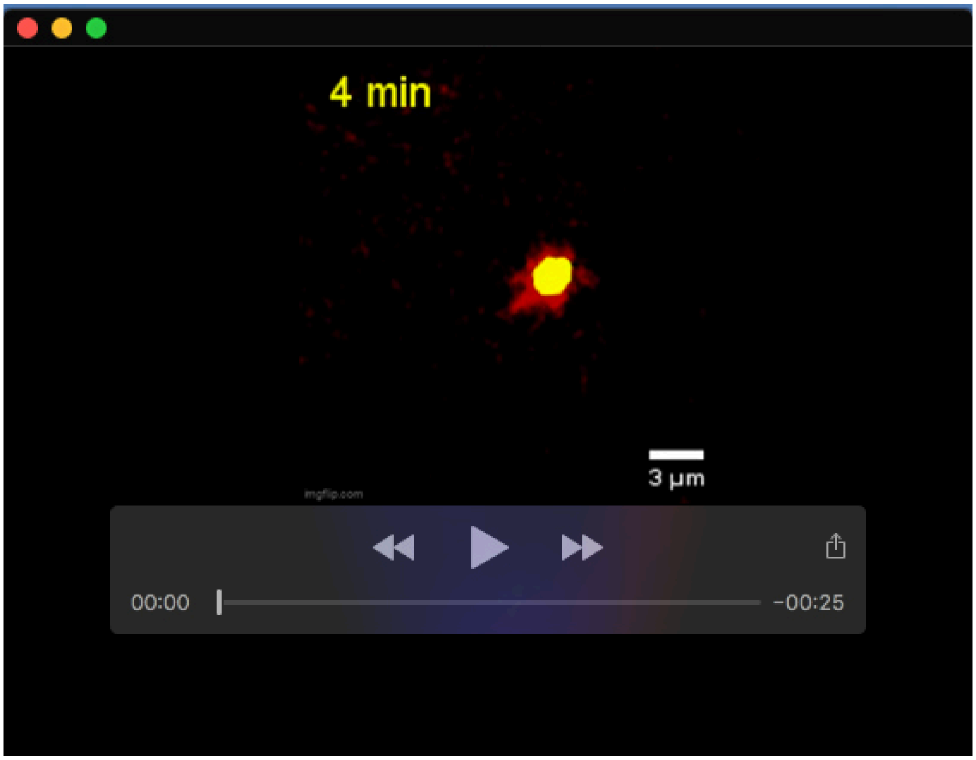

Movie 1. Microtubule aster formation from CDK5RAP2–γ-TuRC assemblies. GFP::CDK5RAP2 scaffolds (green) were incubated with BFP::γ-TuRC, and HiLyte647-labeled tubulin (red) and imaged every 2 min for 30 min at 37°C. As in Fig. 3D.

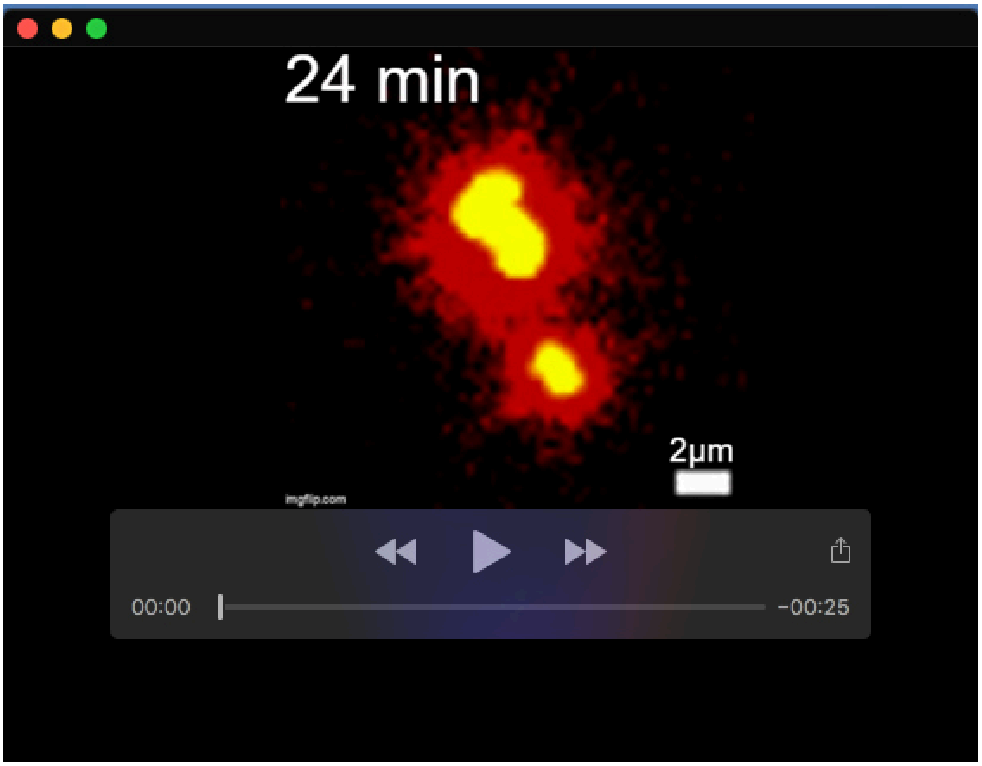

Movie 2. Movement of reconstituted microtubule asters in the presence of HSET and ATP. GFP::CDK5RAP2 scaffolds (green) were incubated with BFP::γ-TuRC, HSET, ATP, and HiLyte647-labeled tubulin (red). As in Fig. 4F.

Journal of Cell Science • Supplementary information
