# Supplementary material for: In vitro reconstitution of a minimal human centrosome scaffold capable of forming and clustering microtubule asters
Source: J Cell Sci. 2025 Jun 27;138(12):jcs264121. doi: 10.1242/jcs.264121 (PMC12273628; doi:10.1242/jcs.264121)
Supplement: Supplementary file 1 [file joces-138-264121_review_history.pdf]

In vitro reconstitution of a minimal human centrosome scaffold capable of forming and clustering microtubule asters

Manolo U. Rios, Weronika E. Stachera, Nicole E. Familiari, Cláudia Brito, Thomas Surrey and Jeffrey B. Woodruff

DOI: 10.1242/jcs.264121

Editor: Renata Basto

Review timeline

Submission to Review Commons:	19 February 2025
Submission to Journal of Cell Science:	30 April 2025
Editorial decision:	12 May 2025
First revision received:	16 May 2025
Accepted:	23 May 2025

Reviewer 1

Evidence, reproducibility and clarity

The present work investigates the assembly of Cdk5rap2 as well as its potential to recruit gamma-tubulin ring complexes, and the interaction between Cdk5rap2 and the motor protein HSET. Consistent with published findings, it is shown here that multimeric assemblies of Cdk5rap2 in vitro are increased in size by Plk1-dependent phosphorylation.

Interestingly, 16 phosphorylation sites by Plk1 are identified in Cdk5rap2, of which 8 represent novel, previously uncharacterized sites. Unfortunately, a characterization of the sites responsible for the multimerization of Cdk5rap2 is not provided, with the exception of S613, which - on the other hand - has only a minor effect on Cdk5rap2 assembly.

Next, it is shown that assemblies of Cdk5rap2 recruit gamma-tubulin ring complexes and form microtubule asters, and that residue F75 in Cdk5rap2 is necessary for efficient gamma-TuRC recruitment and microtubule nucleation. Again, these data are largely confirmatory of previously published work.

In a final paragraph, the interaction of HSET with Cdk5rap2 is investigated. An interaction of the two proteins has been published earlier. As a novel finding, the authors present here that the presence of HSET increases the recruitment of gamma-TuRCs by Cdk5rap2 assemblies. Moreover, it is shown that HSET has the ability to attract alpha/beta-tubulin to Cdk5rap2 assemblies, even in the absence of gamma-TuRCs. Lastly, it is shown that HSET can cluster Cdk5rap2-assemblies in an ATP-dependent manner, as expected from earlier publications on HSET-dependent clustering of centrosomes.

Overall, the manuscript provides novel information on the mechanisms of Cdk5rap2-dependent microtubule nucleation in vitro, as outlined above, but many of the presented data are confirmations of earlier findings. Nevertheless, the information should be of interest to other scientists working in the same field.

On the downside, the claim that multimeric assemblies of Cdk5rap2 represent minimal human centrosomes (see title and abstract) seems exaggerated and not rigorously supported by the data.

1. Centrosomes are self-replicating organelles, whereas multimers of Cdk5rap2 are not. For this reason, the title of the manuscript seems inappropriate to me and should be changed.
2. It is not clear whether the multimers of Cdk5rap2, as produced in vitro, are representative of pericentriolar Cdk5rap2. Whether a scaffold of Cdk5rap2 multimers is formed in vivo, and whether the multimerization is essential for gamma-TuRC activation needs proof. In this context, Haren et al (2009, PLoS One 4:e5976) show Plk1-dependent increase of centrosomal recruitment

© 2025. Published by The Company of Biologists under the terms of the Creative Commons Attribution License (https://creativecommons.org/licenses/by/4.0/).

of Cdk5rap2, but don't address whether this increase is due to multimerization of the protein. The importance of Cdk5rap2 multimerization in microtubule nucleation could be addressed in the authors' in-vitro-system: gamma-TuRC activation and microtubule nucleation could be compared between IgG-bound Cdk5rap2 and IgM-bound (pentameric) Cdk5rap2, or activities could be compared between Plk1-activated and non-activated IgM-dependent Cdk5rap2 assemblies.
3. The role of the different Plk1-dependent phosphorylation sites in the assembly and multimerization of Cdk5rap2 could be tested in more detail.

Minor point:

Parts of the figures are labeled with a font that is difficult to read (in my opinion). The font used here produces very small lower-case letters, but very large capital letters. Could the authors use a more homogeneous font, such as Arial or Calibri, to improve readability?

Significance

As described above in more detail, the manuscript provides novel information on the mechanisms of Cdk5rap2-dependent microtubule nucleation in vitro, but many of the presented data are confirmations of earlier findings.

The information should be of interest to other scientists working in the field of microtubule nucleation and centrosome assembly.

Reviewer 2

Evidence, reproducibility and clarity

In this manuscript, the authors present a novel in vitro reconstitution system using purifiedhuman CDK5RAP2 proteins. They demonstrate that under appropriate conditions, purifiedCDK5RAP2 can self-assemble into micron-scale scaffolds. Using this system, they furthershow that PLK-1 enhances the full-scale self-assembly of CDK5RAP2. These assembliesrecruit and activate γ-TuRCs, leading to microtubule aster formation. Additionally,CDK5RAP2 scaffolds selectively recruit the molecular motor KifC1/HSET, which facilitates α/β tubulin concentration, microtubule polymerization, and clustering of the assemblies.

The main strength of this study lies in the establishment of an in vitro reconstitution systemusing purified human centrosome proteins, including CDK5RAP2, which is over 200 kDa insize. The authors provide evidence that the purified CDK5RAP2 is biologically active, as itperforms expected functions such as γ-TuRC recruitment and microtubule aster formation.However, the novelty of the finding is somewhat limited, as some of the interactionsdescribed have been previously reported. Despite this, the reconstitution system could serveas a valuable tool for investigating regulatory mechanisms of human microtubuleorganization. The manuscript is well-organized and clearly written. Below are specific pointsfor consideration.

1. Functional relevance of CDK5RAP2 in centrosomal microtubule organization. WhileCDK5RAP2 is a major PCM component, its contribution in microtubule organization at thehuman centrosome appears to be more limited compared to its homologs in other species.CDK5RAP2 KO cells do not exhibit major interphase defects, and the majority of mitoticCDK5RAP2 KO cells still form bipolar spindles with organized microtubules at the spindlepoles, albeit with some defects. Additionally, CDK5RAP2 mutant mice display diversephenotypes, including microcephaly, but are not embryonically lethal. In contrast, spd-5mutants in C. elegans and Cnn mutants in D. melanogaster cause embryonic lethality,underscoring their essential role in mitosis and cell survival. As the authors note, the humancentrosome requires additional PCM components for fully functional microtubuleorganization. The CDK5RAP2 scaffolds described in this manuscript likely function asmicrotubule-organizing centers both within and outside the centrosome. Given this, thephrase "minimal human centrosomes" in the title may overstate the findings and should be revised.
2. Dynamics of CDK5RAP2 assembly. It would be valuable to investigate the dynamics ofCDK5RAP2 scaffold assembly and disassembly in vitro. Do GFP::CDK5RAP2 scaffolds fuseupon the addition of IgM and PEG? Do GFP::CDK5RAP2 scaffolds disassemble upondilution? Do CDK5RAP2 scaffolds exhibit

© 2025. Published by The Company of Biologists under the terms of the Creative Commons Attribution License (https://creativecommons.org/licenses/by/4.0/).

rapid fluorescence recover after photobleaching?Addressing these questions would provide insight into the biophysical properties ofCDK5RAP2 assemblies and their regulation.3.
3.Figure 2A: Clarify why 720 nM GFP::CDK5RAP2 was specifically chosen for this group.Does the addition of PLK-1 inhibit CDK5RAP2 assembly?

The main strength of this study lies in the establishment of an in vitro reconstitution systemusing purified human centrosome proteins, including CDK5RAP2, which is over 200 kDa insize. The authors provide evidence that the purified CDK5RAP2 is biologically active, as itperforms expected functions such as γ-TuRC recruitment and microtubule aster formation.However, the novelty of the finding is somewhat limited, as some of the interactionsdescribed have been previously reported. Despite this, the reconstitution system could serveas a valuable tool for investigating regulatory mechanisms of human microtubuleorganization. The manuscript is well-organized and clearly written.

Reviewer 3

Evidence, reproducibility and clarity

This paper develops an in vitro minimal component system to study PCM assembly and microtubule organisation. They purify the key centrosomal protein CDK5RAP2 and show that it forms micron-sized assemblies in vitro either when crowing agents are applied or when an antibody seed is used (the pentameric IgM antibody targets the CM2 domain at the C-term of CDK5RAP2). Addition of Plk1 kinase phosphorylates CDK5RAP2 on multiple sites and moderately increases assembly size. The authors show that these assemblies are inherently unable to nucleate microtubules unless purified γ-TuRCs or γ-TuRCs+HSET are added.
Mutation of the well-known F75 residue largely prevents γ-TuRC recruitment, as expected. The minus-end-directed motor HSET, known to cluster centrosomes in vivo, can also be recruited to the assemblies via its N-terminal IDR domain. HSET enhances γ-TuRC recruitment and microtubule aster formation and allows assemblies to better connect via their asters.

Overall, this is a nice, albeit short, story that provides a platform for future bottom-up experiments to probe the mechanism of PCM assembly and microtubule nucleation. Nevertheless, the authors could have performed some additional experiments to make better use of the system and to generate some more robust conclusions. I feel some conclusions are not fully supported by the data. I outline these points in more detail below.

Major comments

This paper develops an in vitro minimal component system to study PCM assembly and microtubule organisation. They purify the key centrosomal protein CDK5RAP2 and show that it forms micron-sized assemblies in vitro either when crowing agents are applied or when an antibody seed is used (the pentameric IgM antibody targets the CM2 domain at the C-term of CDK5RAP2). Addition of Plk1 kinase phosphorylates CDK5RAP2 on multiple sites and moderately increases assembly size. The authors show that these assemblies are inherently unable to nucleate microtubules unless purified γ-TuRCs or γ-TuRCs+HSET are added.
Mutation of the well-known F75 residue largely prevents γ-TuRC recruitment, as expected. The minus-end-directed motor HSET, known to cluster centrosomes in vivo, can also be recruited to the assemblies via its N-terminal IDR domain. HSET enhances γ-TuRC recruitment and microtubule aster formation and allows assemblies to better connect via their asters.

Overall, this is a nice, albeit short, story that provides a platform for future bottom-up experiments to probe the mechanism of PCM assembly and microtubule nucleation. Nevertheless, the authors could have performed some additional experiments to make better use of the system and to generate some more robust conclusions. I feel some conclusions are not fully supported by the data. I outline these points in more detail below.

Major comments

1. I think the authors have to be a bit careful in their comparisons with what happens in cells. There appear to be some differences. For example, they show that micron-sized assemblies of

© 2025. Published by The Company of Biologists under the terms of the Creative Commons Attribution License (https://creativecommons.org/licenses/by/4.0/).

CDK5RAP2 form when using the IgM antibody as a seed. But it would seem more likely that the IgM-induced assemblies would mimic the situation in interphase, where CDK5RAP2 forms a thinner layer around the centriole. In the assemblies in vitro, CDK5RAP2 molecules must be self-associating such that they can spread relatively far from the IgM antibody. In vivo, this occurs only in mitosis and is probably induced by phosphorylation and the presence of other stabilising factors, such as Pericentrin and CEP192. So why do the in vitro assemblies spread so far in vitro? I assume it must be that there is a propensity for CDK5RAP2 to self-associate and this propensity is enhanced when other proteins are not present (that may otherwise bind to CDK5RAP2 and prevent CDK5RAP2 from self- associating unpacifically. I think this should be discussed and the conclusions should be toned down regarding how the assemblies recapitulate what occurs in vivo.

2. The purified proteins used in the experiments should be analysed using size exclusion or SEC-MALS or other analytical techniques to check that they behave well and do not aggregate.

3. When statistically comparing the size of assemblies in Figure 2B,E the authors need to take into account that the data does not look normally distributed (this can be tested) and so they should not be using a t-test, unless they first transform the data e.g. log transform, such that the data is normally distributed. If they can't transform the data, they need to use a non- parametric test. This is particularly important for Figure 2E, where the difference is very small. Moreover, because the N number is so high, it is likely that only a small, non- physiologically meaningful difference, may appear statistically significant. Thus, the conclusion that the S613E mutant promotes assembly growth may be wrong. Given the wide variation in assembly size and issues with high N, it may be more prudent to calculate the average assembly size per experiment and then repeat the experiment multiple times, plotting the average value for each experiment. The data will then be normally distributed and less variable and the issue with having such a high N will disappear.

4. The authors claim that their "minimal module of self-assembled CDK5RAP2, γ- TuRCs, and a/b tubulin is sufficient to recapitulate microtubule aster formation", but it's difficult to say whether microtubule aster formation (as seen in cells) is being recapitulated in this experiment. It is very possible that the MT asters seen in vitro are not as robust as those organised by centrosomes in vivo. It would be more appropriate to say that a minimal system of self-assembled CDK5RAP2, g-turc and tubulin is sufficient to allow aster formation, although whether these asters are as robust as those observed within cells is not clear.

5. The authors could significantly strengthen their claim that Plk1 phosphorylation of CDK5RAP2 promotes assembly formation by testing the other phosphorylation sites. It seemed strange to choose only one to test. It would seem sensible to try a phospho-mimetic mutant that contains mutations at each of the 16 sites and presumably show that this strongly increases assembly formation. This would help rule out that Plk1 promotes assembly formation simply by binding to CDK5RAP2. It would also be interesting to test different combinations of mutants e.g. N-term sites, mid sites and c-term sites, or sites in CC versus sites in disordered regions.

6. In Figure S3C, the authors show no correlation between γ-TuRC and a/ß-tubulin levels in F75A assemblies and so conclude that the bound γ-TuRCs were incompetent to bind a/b tubulin, unlike the control case, They therefore conclude that F75 is required for activating the γ-TuRC but is not absolutely required for γ-TuRC binding. They therefore propose "microtubule aster formation involves a two-step process of γ-TuRC binding to CDK5RAP2 and subsequent activation via interactions mediated by F75". I think this conclusion is not supported by the data. The lack of correlation observed in Fig S3C is probably due to the very low levels of γ-TuRC recruitment and the noise in the data. If the authors tried to use similar γ-TuRC levels in the wild-type condition to make a correlation with a/ß-tubulin levels, I suspect they would not observe one either i.e. take only those wild-type assemblies that have <2.2 units of γ-TuRC see if there is a correlation with a/ß-tubulin levels. From the graph in FigS3B there does not seem to be a correlation in this region of the graph. Thus, there is no evidence for a two-step mechanism. It is much more likely that binding of the helix containing F75 both allows γ-TuRC to dock to the centrosome and induces conformational changes that activate the γ-TuRC at the same time, which would be a one-step process. The residual amount of γ-TuRC the authors observe in the F75 mutant is likely due to unspecific binding of a large multi-protein complex to a multi-valent protein that is not in its native environment. These unspecific binding sites would likely be covered by other proteins in the context of a centrosome. In line with this comment, the authors should change the word "activation" on line 295 to "recruitment" and adjust what they say on lines 332-333.

© 2025. Published by The Company of Biologists under the terms of the Creative Commons Attribution License (https://creativecommons.org/licenses/by/4.0/).

7. Lines 267-269: "We hypothesize HSET can potentiate CDK5RAP2/γ-TuRC aster- formation capabilities through increased a/b tubulin and γ-TuRC partitioning and perhaps via γ- TuRC activation". There is no evidence for γ-TuRC activation so that part should be removed.
8. The authors claim that the system recapitulates centrosome clustering when HSET is added, but as the authors admit the clustering of assemblies is not very good ("Once the microtubule bridges had formed, we did not observe further motor-mediated constriction of CDK5RAP2 assemblies (Supplemental Movie 2"). This should be toned down.
9. When the authors talk about the ability of HSET to recruit a/ß-tubulin to assemblies, the authors should discuss this in light of the findings of Norris 2018 that showed how HSET can bind soluble tubulin. This result is therefore not that unexpected.
10. The proposed model for PCM assembly (lines 301-307) is an over-simplification. The centrosome scaffold will involve interactions with Pericentrin and CEP192 that the current work does not take into account. I know the authors mention this later, but this paragraph is still misleading when taken in isolation.

Minor comments

1. Lines 136-137: "CDK5RAP2 naturally docks with the centriole indirectly via its CM2 domain (Wang et al., 2010)." I think this paper showed the CM2 domain was necessary for localisation to centrosomes but not that it was a docking domain, as suggested by this sentence. The role of the CM2 domain could instead bye to allow CDK5RAP2 to assemble into a scaffold by interacting with other parts of CDK5RAP2, which is in turn necessary for centrosome localisation, as is the case for fly Cnn (CM2-PReM interaction).
2. Lines 139-140: "We hypothesized that by using an antibody that closely recapitulates the geometry, valence, and scale of centrioles". How do you know a centriole has a valency of 5? Is an IgM antibody of the same diameter as a centriole?
3. lines 196-198: "Our results suggest that phosphorylation of CDK5RAP2 promotes its multimerization in the absence of centrioles, similar to the mechanism proposed for SPD-5 (C. elegans) (Rios et al., 2024; Woodruff et al., 2015)". This is also true of fly Cnn, as phospho-mimetic Cnn forms assemblies in the cytosol in the absence of centrosomes (Conduit 2014).
4. Lines 205-208: "In vivo, CDK5RAP2 recruits γ-TuRCs to produce robust microtubule asters, giving centrosomes their main function (Choi et al., 2010; Fong et al., 2008). However, it is not known if CDK5RAP2 and γ-TuRCs are sufficient to achieve robust microtubule aster formation or if additional components are required." There is good evidence that g-TuRC recruitment is more dependent on NEDD1 than on CDK5RAP2 e.g. Haren 2009. This fact does not seem to be taken into account in these two sentences.
5. The authors speculate that "HSET's intrinsic aster-formation capabilities are hindered by its interaction with the CDK5RAP2 scaffold, presumably due to preferential binding of CDK5RAP2 for HSET's IDR". Could it also be that the concentration of tubulin was not high enough to form MTs in the absence of γ-TuRCs?

Optional experiments

1. The authors use a wild-type form of CDK5RAP2 that in theory should be auto-inhibited from binding γ-TuRCs, at least to some degree (based on the findings from Yang 2023). The authors could consider introducing mutations into CDK5RAP2 that would relieve the reported auto-inhibition, such as the phosphomimetic mutations used in Yang et al., 2023. In theory, this would allow more g-turc recruitment than seen in WT and help better recapitulate the situation in cells. At the minimum, auto-inhibition should be discussed, pointing out that CDK5RAP2 cannot be fully auto-inhibited from binding γ-TuRCs, at least under the conditions used in the experiments.
2. Given that HSET appears to help recruit γ-TuRCs, the authors could test whether purified HSET binds to purified γ-TuRCs

Studies in flies and worms have shown that a the large coiled coil proteins, Cnn and SPD-5, assemble into centrosomal scaffolds in a phosphorylation/Plk1-dependent manner and that these scaffolds support overall PCM assembly during mitosis. CDK5RAP2 is the proposed human homologue of Cnn and SPD-5 because it is similarly large and coiled-coiled and because it contains the N-terminal γ-TuRC binding CM1 domain and the C-terminal centrosome localising CM2 domain. Depletion experiments in human cells have shown that CDK5RAP2 is also required for proper mitotic PCM assembly. Nevertheless, it remains unclear whether CDK5RAP2 also self-assembles

© 2025. Published by The Company of Biologists under the terms of the Creative Commons Attribution License (https://creativecommons.org/licenses/by/4.0/).

into scaffold that supports PCM assembly. The authors do a great job in purifying full-length CDK5RAP2 and making assemblies using an antibody seed rather than using crowing agents (which has been questioned as a good way to recapitulate the in vivo situation). The data presented is consistent with the idea that CDK5RAP2 can form centrosome scaffolds that can recruit other factors, such as γ-TuRCs and there is evidence that this is promoted by Plk1. The data is therefore consistent with CDK5RAP2 being able to self-associate to form a scaffold important for PCM assembly. It must be noted, however, that the scaffolding in vitro does not exactly mimic what happens in vivo (e.g. micron-sized scaffold assemblies form in vitro without the need for phosphorylation or other proteins such as Pericentrin). The results on F75 are expected, as this residue is known to allow CDK5RAP2 to bind γ-TuRCs when N-terminal fragments are used in IP experiments, and I don't agree with the conclusion that there is a two-step mechanism of binding and then activation (see above). The data that HSET increases the recruitment of γ- TuRCs and tubulin is interesting, but the molecular detail for the γ-TuRC recruitment is lacking. Overall, we have learnt something, but the data does not provide a large advance in our understanding of centrosome biology. I feel the authors could take better advantage of the neat system they have now established. They mention in the discussion how the setup could be used to better understand centrosome biology and so it seems crying out to use it to a greater extent e.g. adding in other key PCM proteins such as pericentrin, cep192, aurA...etc.

Original submission

<u>First decision letter</u>

MS ID#: jcs.264121

MS TITLE: In vitro reconstitution of a minimal human centrosome scaffold capable of forming and clustering microtubule asters

AUTHORS: Manolo Uriel Rios; Weronika Stachera; Nicole Familiari; ClÃ¡udia Brito; Thomas Surrey; Jeffrey Woodruff

ARTICLE TYPE: Review Commons Transfer

Dear Jeff,

Thank you for transferring your article to JCS. It has now been seen by one of the original reviewers.

To see the reviewers' reports and a copy of this decision letter, please go to:

As you will see, this reviewer gave favourable reports but raised some critical points that will require amendments to your manuscript. I hope that you will be able to carry these out because I would like to be able to accept your paper.

First revision

<u>Author response to reviewers' comments</u>

Point by point response

Reviewer 1: The authors have done a reasonable job at addressing my concerns, although I still have a few points that need addressing. The most important being about whether there is a two-step mechanism or not. Also, just a note for future rebuttals, it would have been helpful if the authors could have included the updated text within the rebuttal, or at least cite the lines in the

© 2025. Published by The Company of Biologists under the terms of the Creative Commons Attribution License (https://creativecommons.org/licenses/by/4.0/).

paper, so that the Reviewer doesn't need to search for them in the paper. For example, just saying that "we have updated the discussion to highlight this point" is not that helpful.

We apologize for this error. We have now included any revised text in this response letter.

Original point 3: This was about the use of statistics and the authors reassessed the data and found "the differences between WT and S613E are not significant". But in the main text they say they p value is <0.02…? In the Figure, they say "n.s.". I'm assuming the p<0.02 is a mistake….? I also have an issue with their conclusion. They say "This result suggests that phosphorylation of residue S613 does not significantly enhance growth of CDK5RAP2 assemblies. Since this does not reach the effect of adding PLK-1(CA), additional phosphorylations are needed to achieve full-scale scaffold size." Saying that additional phosphorylations are needed implies that phosphorylation of S613 is actually doing something. But for this they would need to show that mixing an S613A mutant with Plk1 reduces the size of scaffolds compared to mixing the WT CDK5RAP2. As it stands, there is no evidence that S613 phosphorylation is contributing to scaffold formation.

We thank the reviewer for pointing out the error with the P value. Indeed, the value is non-significant (P=0.3). We have corrected the main text.

Moreover, we agree with the reviewer's point about the importance of S613 for scaffold growth. We have changed the text accordingly to remove the statement implying S613 is one of many sites necessary for scaffold size:

"The reaction contained the anti-CM2 antibody but no PLK-1 or ATP·$MgCl_2$. Individual GFP::CDK5RAP2(S613E) assemblies were, on average, 1.1-fold more massive than WT assemblies (P=0.3)(**Fig. 2E, F**). This result suggests that phosphorylation of residue S613 does not significantly enhance growth of CDK5RAP2 assemblies. However, we observed that S613E formed twice as many assemblies compared with WT (P<0.0001)(Fig. 2G). This suggests that S613E is sufficient to promote CDK5RAP2 nucleation when using a synthetic nucleating seed."

Original point 6: This point relates to whether or not there is a two-step mechanism for γ-TuRC recruitment and activation by CDK5RAP2. I still think that there is no strong evidence for a two-step mechanism. First, I think the authors misunderstood the analysis I was suggesting. I was suggesting that they perform a correlation analysis of the low recruitment WT assemblies i.e. is there a correlation between γ-TuRC partitioning and tubulin partitioning at WT assemblies that have a γ-TuRC partitioning score <2.2 ? I suspect there will not be due to the noise in the data. They should therefore not expect a correlation between γ-TuRC partitioning and tubulin partitioning at F75A assemblies because the γ-TuRC partitioning scores are all <2.2, meaning that the signal is too close to noise levels to obtain a correlation. For me, this is a really important point as the lack of correlation is being used to help conclude that there is a 2-step mechanism for recruitment and activation. In reality, the minimal amount of low-activity γ-TuRC recruited in the F75A mutant is likely irrelevant for two reasons: 1) there is only a minimal amount recruited meaning that the vast majority of γ-TuRC recruited to centrosomes by WT CDK5RAP2 is via the CM1 domain, which will activate the γ-TuRCs on binding i.e. a one-step mechanism; 2) the minimal amount of γ-TuRC recruited to F75A scaffolds could be an artefact of the in vitro system i.e. the γ-TuRCs are binding to sites on CDK5RAP2 that would normally be interacting with other centrosomal proteins and therefore these sites would be excluded from binding to γ-TuRCs. If the authors really want to stick to their claim that there is a two-step mechanism, I think they need to be clearer that this would be, at best, a very minor part of CDK5RAP2's role in mediating microtubule nucleation from the centrosome (as the majority is via the CM1 binding one-step mechanism of recruitment and activation).

In the discussion, the authors say that "These data indicate that 1) γ-TuRCs can bind to CDK5RAP2 and remain inactive" - whether this is true in vivo remains uncertain, as discussed above. So far, the authors show very minimal binding in an artificial system where unspecific binding is likely due to the absence of other CDK5RAP2 interacting proteins. "2) additional domains beyond CM1 mediate γ-TuRC recruitment" - this should be rephased to acknowledge that the authors do not know this to be true in vivo e.g. additional domains beyond CM1 are capable of mediating minimal γ-TuRC recruitment to in vitro assemblies.

© 2025. Published by The Company of Biologists under the terms of the Creative Commons Attribution License (https://creativecommons.org/licenses/by/4.0/).

We now understand better the reviewer's point, and we apologize for misinterpreting it in the first rebuttal. We performed the correlation analysis as suggested and realize that the reviewer is correct: there is no correlation between γ-TuRC levels and tubulin levels in the narrow partition range of <2.2. We have revised the text to emphasize that F75 is the major site contributing to γ-TuRC binding and activation, and we have removed all mention of a two-step mechanism. Since we are changing our conclusion to be more conservative, we decided not include the correlation analysis as it is a negative result that measures mostly noise. We have changed the main body and discussion:

"This result reinforces the importance of F75 for the interaction between γ-TuRC and CDK5RAP2 and demonstrates that our system recapitulates binding specificity. The fact that the F75A mutant still recruits minute amounts of γ-TuRC could be an artifact of this system. Alternatively, it could suggest that additional less-important interaction motifs besides CM1 could engage with the γ-TuRC. In these mutant assemblies, there was no correlation between γ-TuRC and □/□ tubulin recruitment, indicating that the bound γ-TuRCs were incompetent to bind □/□ tubulin, unlike the control case (**Fig. S3C**). Even in the low γ-TuRC partitioning range, GFP::CDK5RAP2(WT) scaffolds significantly recruit more □/□□tubulin than the F75 mutant (P<0.0001)(**Fig. S3D**). Consistently, F75A assemblies did not nucleate microtubule asters (0%, n = 490 assemblies) (**Fig. 3A**). Thus, F75 is required for recruiting the γ-TuRC and improving its ability to bind □/□ tubulin."

"This result suggested that CDK5RAP2 recruits γ-TuRC through its CM1 domain. Whether F75 is critical in the context of full-length CDK5RAP2 had not been tested. We saw that assemblies made with full-length, wild-type CDK5RAP2 could strongly recruit γ-TuRC while the F75A mutant could not. Furthermore, γ-TuRCs were only active when bound to wild-type CDK5RAP2. These data indicate that CM1 is the primary mediator of γ-TuRC recruitment and that F75 is critical for activating γ-TuRCs, likely due to mediating ring closure (Xu et al., 2024)."

Original point 8: Regarding the HSET allowing assemblies to cluster. I originally pointed out that the assemblies did not fully cluster and remained separated. The authors now highlight this in the results but they still claim that the "results demonstrate that our system recapitulates HSET binding specificity and centrosome clustering in vitro". I think this is still an overstatement as clustering is not fully recapitulated. They should replace their statement by what they say in the rebuttal i.e. "This suggests that we have recapitulated a key aspect of centrosome clustering", the key aspect being bringing the assemblies closer together. But in vivo, the clustered centrosomes do not remain separated by microtubule bridges, so they have not properly recapitulated what occurs in vivo.

We updated this as suggested:

"These results demonstrate that our system recapitulates HSET binding specificity and a key aspect of centrosome clustering."

<u>Second decision letter</u>

MS ID#: jcs.264121R1

MS Title: In vitro reconstitution of a minimal human centrosome scaffold capable of forming and clustering microtubule asters

Authors: Manolo Uriel Rios; Weronika Stachera; Nicole Familiari; ClÃ¡udia Brito; Thomas Surrey; Jeffrey Woodruff

Article Type: Review Commons Transfer

Dear Dr Woodruff,

I am happy to tell you that your manuscript has been accepted for publication in Journal of Cell Science, pending standard publication integrity checks.

Thank you for sending your manuscript to Journal of Cell Science through Review Commons.

© 2025. Published by The Company of Biologists under the terms of the Creative Commons Attribution License (https://creativecommons.org/licenses/by/4.0/).
